# Single-Cell RNA Sequencing Reveals the Immunological Profiles of Renal Allograft Rejection in Mice

**DOI:** 10.3389/fimmu.2021.693608

**Published:** 2021-07-22

**Authors:** Qixia Shen, Yucheng Wang, Jiaoyi Chen, Lifeng Ma, Xiaoru Huang, Sydney C. W. Tang, Huiyao Lan, Hong Jiang, Jianghua Chen

**Affiliations:** ^1^ Kidney Disease Center, The First Affiliated Hospital, College of Medicine, Zhejiang University, Hangzhou, China; ^2^ Key Laboratory of Nephropathy, The First Affiliated Hospital, College of Medicine, Zhejiang University, Hangzhou, China; ^3^ Institute of Nephrology, Zhejiang University, Hangzhou, China; ^4^ Department of Medicine & Therapeutics and Li Ka Shing Institute of Health Sciences, The Chinese University of Hong Kong, Hong Kong, China; ^5^ Center for Stem Cell and Regenerative Medicine, The First Affiliated Hospital, College of Medicine, Zhejiang University, Hangzhou, China; ^6^ Guangdong-Hong Kong Joint Laboratory on Immunological and Genetic Kidney Diseases, Guangdong Academy of Medical Sciences, Guangdong Provincial People’s Hospital, Guangzhou, China; ^7^ Division of Nephrology, Department of Medicine, The University of Hong Kong, Hong Kong, China

**Keywords:** ScRNA-seq, immunological profiles, renal transplantation, acute rejection, chronic rejection

## Abstract

Allograft rejection is a common immunological feature in renal transplantation and is associated with reduced graft survival. A mouse renal allograft rejection model was induced and single-cell RNA sequencing (scRNA-seq) data of CD45^+^ leukocytes in kidney allografts on days 7 (D7) and 15 (D15) after operation was analyzed to reveal a full immunological profiling. We identified 20 immune cell types among 10,921 leukocytes. Macrophages and CD8^+^ T cells constituted the main populations on both timepoints. In the process from acute rejection (AR) towards chronic rejection (CR), the proportion of proliferating and naïve CD8^+^ T cells dropped significantly. Both B cells and neutrophils decreased by about 3 folds. On the contrary, the proportion of macrophages and dendritic cells (DCs) increased significantly, especially by about a 4.5-fold increase in Ly6c^lo^Mrc1^+^ macrophages and 2.6 folds increase in Ly6c^lo^Ear2^+^ macrophages. Moreover, myeloid cells harbored the richest ligand and receptor (LR) pairs with other cells, particularly for chemokine ligands such as Cxcl9, Cxcl10, Cxcl16 and Yars. However, macrophages with weak response to interferon gamma (IFNg) contributed to rejection chronicization. To conclude, reduction in CD8 T cells, B cells and neutrophils while increasing in Ly6c^lo^Mrc1^+^ macrophages and Ly6c^lo^Ear2^+^ macrophages, may contribute significantly to the progress from AR towards CR.

## Introduction

Rejection of the transplanted kidney in humans is still a major cause of morbidity and mortality. Despite current immunosuppressive therapy, AR develops in about 10–12% of transplant patients (1). Besides, the long-term survival rate is not satisfactory, as nearly half of the transplanted kidney function gradually loses within 10 years (2). scRNA-seq has enabled the profiling of specific cell populations at the single-cell level, offering an unprecedented opportunity to define cell types and states comprehensively with molecular precision. However, due to unpredictability of tissue availability, limited size of kidney biopsy samples, challenges are raised in wide application of scRNA-seq to dissect immunological network and dynamic changes involved in human kidney allograft rejection. The mouse genome has been well characterized and the protein-coding regions of mouse and human genomes are 85% identical (3). Thus, mouse models of diseases are available for scRNA-seq to reveal the characteristics of immunity (4–6). Of them, the fully mismatched mouse model of renal transplantation is a better model that closely relates to that of human both technically and pathologically (7). Studying the evolution of the pathology of rejection in mouse kidney allografts, interstitial infiltrate, major histocompatibility complex (MHC) induction, and venulitis peaked at day 7-10, then stabilized or regressed by day 21 (8). By scRNA-seq, immune cells isolated from allografts undergoing AR on D7 to CR on D15 following kidney transplantation should gain new insights into the immunological profiling, dynamic changes from AR to CR during renal transplant rejection.

## Materials And Methods

### Mouse Kidney Transplantation

Ectopic renal transplantation was performed by transplanting kidneys from male BALB/c mice (H‐2d; 8 weeks old) into male C57Bl6/J recipients (H‐2b; 8 weeks old) as previously described (9, 10). Mice were euthanized on D7 after the operation. Animal studies were performed according to the Department of Health (Hong Kong) guidelines in the Care and Use of Animals and the experimental protocol was approved by the Animal Experimentation Ethics Committee at the Chinese University of Hong Kong.

### Histology

Kidney tissues were fixed in formalin and embedded in paraffin, sectioned into 3 µm slices for hemotoxylin & eosin (HE), periodic acid–Schiff (PAS) and Masson staining.

### Serum Enzyme-Linked ImmunoSorbent Assay (ELISA)

Blood was collected in serum separator tube by cardiac puncture from WT C57Bl6/J mice, kidney allograft recipients sacrificed on D7 and D15 after kidney transplantation. Samples were allowed to clot for 30 min at room temperature before centrifugation for 15 min at 1,000***g***, 4°C. Remove serum, aliquot, and store samples at −80°C. Serum IgG and IgM ELISA (Cat# ab157719, ab215085; Abcam, Quantikine, USA) was performed as per the manufacturers’ recommendations.

### Fluorescence-Activated Cell Sorting (FACS)

After sacrifice, mice were flashed with 0.9% saline through heart to remove the peripheral blood remained in the kidney allografts. Kidney graft tissues were then collected and digested using Blenzyme 4 (Roche Inc., Indianapolis, IN). Kidney suspensions were pooled and stained with Live/dead Aqua Fluorescent Reactive Dye (Life Technologies) and PE conjugated anti-mouse CD45 antibody (eBioscience). DAPI^-^CD45^+^ cells were FACS-sorted on (BD Influx™ cell sorter) with a 100 µm nozzle and collected (**Figure S1A**).

### A 10× Sample Processing, cDNA Library Preparation and Single Cell Mapping

Cells were assessed for viability and counted using Countess II FL Automated Cell Counter, loaded into individual wells of 10× Chromium Single Cell chip. Single cells were then encapsulated into Gel in-beads emulsions by 10× Chromium Controller. Chromium™ Single Cell 5’ Reagent Kits (v1 chemistry) were used to perform reverse transcription on Gel in-beads emulsions, followed by cDNA clean up and amplification. The double-stranded cDNA was then gone through enzymatic fragmentation, adapter ligation, index PCR and SPRI select size selection per manufacturer’s protocol. Library size and concentrations were determined by Qubit, quantitative PCR and Bioanalyzer assays. scRNA-seq data were mapped to the mouse (mm10) reference genome and quantified using CellRanger 2.2.0 supplied by 10×.

### D15 CD45^+^ Leukocytes Extraction and Data Sets Integration

D15 data sets, GSM4761000 and GSM4761003, contained in dataset GSE157292 were downloaded from the Gene Expression Omnibus (GEO). Cells from both D7 and D15 datasets were included with the exclusion criteria (1): cells with greater than 6% mitochondrial genes (2), cells expressing less than 200 genes or greater than 5,000 genes, and (3) cells with less than 1,000 unique molecular identifier (UMI) counts or greater than 40,000 UMI counts. All these cells were removed to filter out low-quality cells and doublets. Next, Uniform Manifold Approximation and Projection (UMAP) were used for dimension reduction and clustering by Seurat 3.0 version on dataset GSE157292. Cell clusters expressing gene Ptprc (cluster 0:7, 9, 11, 12, 15:19) were then extracted as D15 dataset (**Supplementary Figure 2**). Normalization, feature selection, scaling and principal component analysis were then performed separately for D7 and D15 datasets before integration. Again, UMAP were used for dimension reduction and clustering.

### Single Cell Marker Identification and Differential Expression

Marker genes were determined for each cluster by Wilcoxon Rank Sum test within the FindAllMarkers function using genes expressed in a minimum of 10% of cells and average natural log‐transformed fold change threshold of 0.25. We defined a gene to be a cluster-specific marker if it was differentially up-regulated in a cluster as compared to the remaining clusters. To identify differential expression genes (DEGs), we used Seurat FindMarkers function using genes expressed in a minimum of 10% of cells and with an adjusted p value (adj_p value) threshold of 0.05.

### Gene Ontology (GO) and Gene Set Variation Analysis (GSVA)

GO enrichment analysis was performed on Metascape (metascape.org), gene-set enrichment score was calculated with GSVA software from Bioconductor (version 1.28). Marker genes or DEGs with average log_2_ fold change (avg_log_2_FC) >0.59, adj_p value <0.05 were used for GO. Gene-sets were downloaded from the Mouse Genome Informatics (http://www.informatics.jax.org/). Raw counts file was used as input data. R/Bioconductor Limma package was used to test whether the gene sets were either upregulated on D7 or D15.

### Single-Cell Regulatory Network Inference and Clustering (SCENIC)

SCENIC was performed following pySCENIC (11). Activity score of each regulon was calculated in each single cell through the area under the recovery curve to evaluate whether a regulon was enriched at the top ranking of cells. Transcription factors (TFs) motif enrichment analysis was then performed to identify the direct targets (regulons) and score the activity of the regulons (AUCell score). AUCell score was then projected onto the UMAP or scaled to plot heatmaps.

### Pseudotime Analysis

The original gene expression matrix of mononuclear/macrophage cell populations (C1, C2, C6, C7, C12 and C16) were used as the input files for creating the Monocle 2.0 CellDataSet. The DDRTree method was used for dimensionality reduction. Batch effect was removed by the residualModelFormulaStr function.

### Ligand–Receptor (LR) Interaction

We conducted cell–cell interaction analysis utilizing cellphonedb function curated by the CellPhoneDB database (12). The significant cell–cell interactions were selected with p value <0.01. Heatmaps of significant interaction pairs were plotted using mean values of interacting partners. Mean value refers to the total mean of the individual partner average expression values in the corresponding interacting pairs of cell types.

### Statistics

Data were expressed as the mean ± SD and analyzed using student t test and one-way analysis of variance, followed by Newman–Keuls multiple comparison test from GraphPad Prism 6.07 (GraphPad Software Inc, San Diego, CA, USA). p  < 0.05 was considered statistically significant.

## Results

### AR Progressed Towards CR From D7 to D15 During Mouse Renal Allograft Rejection

**Figure 1:**
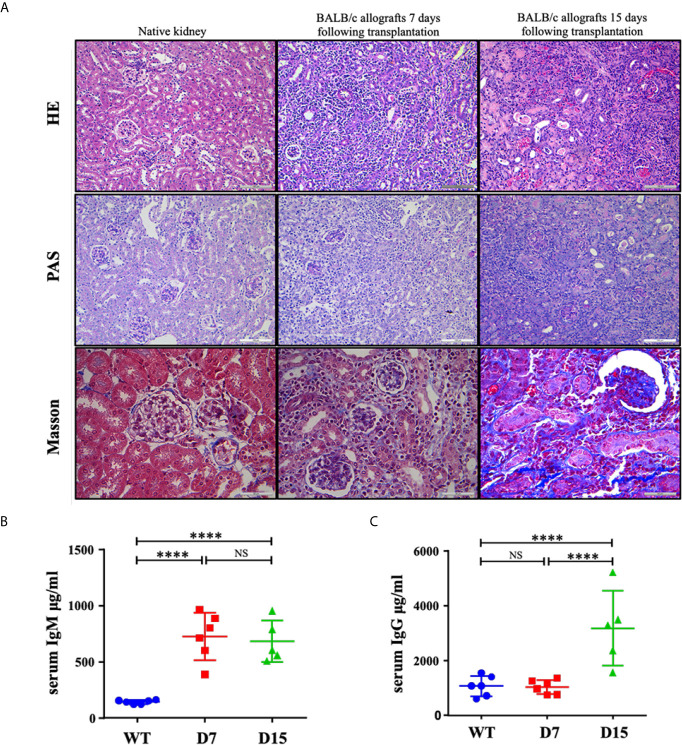
Representative histological tubular injury in the transplanted kidneys. **(A)** Representative pictures of HE, PAS and Masson staining of native kidneys and allografted kidneys. Native kidneys do not exhibit tubular injury. Seven and 15 days following transplantation, BALB/c allografts undergo acute rejection with diffuse mononuclear cell infiltrates, perivascular lymphocytic infiltrates, necrotic tubules and tubulitis. Collagen fibers were only noted in some parts of BALB/c allografts collected 15 days following transplantation as Masson staining showed. ELISA measurements of total **(B)** IgM and **(C)** IgG concentrations in serum from WT C57Bl6/J mice (n = 6), kidney allograft recipients sacrificed on D7 (n = 6) and D15 (n = 5). WT, wild type; D7, 7 days following transplantation; D15, 15 days following transplantation. Data are presented as mean ± SD, and n represents number of mice. ****p < 0.0001. NS, not significant.

After tissue harvesting, histological features of kidney allografts from WT and kidney allograft recipient mice were examined by HE, PAS and Masson staining (**Figure 1A**). Native kidneys do not exhibit tubular injury. Diffuse mononuclear cell infiltrates throughout the renal parenchyma, severe arteritis, necrotic tubules and tubulitis were observed in allografted kidneys on both D7 and D15 following transplantation. However, Masson staining also detected that moderate fibrosis was developed on D15 after the operation, whereas almost no collagen fibers were observed on D7 (**Figure 1A**). Thus, histologic evidence indicated that it was AR on D7 and a mix rejection pattern on D15. Interestingly, although serum levels of IgM were markedly elevated on both 7 and 15 days after kidney transplantation, an increase in serum levels of IgG was only observed on D15 (**Figures 1B, C** **)**, suggesting a switch from the early acute T cell-mediated rejection to the late mixed T cells and antibody-mediated rejection.

### scRNA-Seq Identified Major Immune Cell Types in Mouse Kidney Allografts

**Figure 2:**
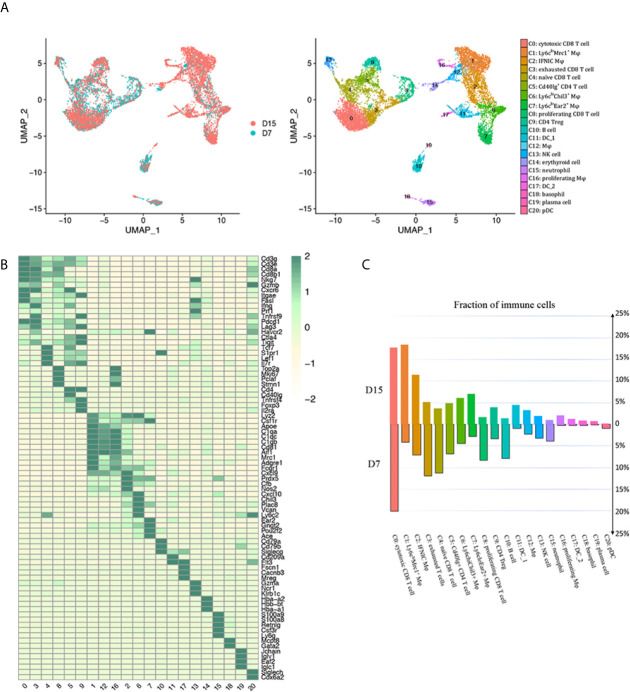
scRNA-seq of mice kidney allograft revealed the presence of 20 CD45^+^ immune cell clusters. **(A)** UMAP plot of cell clusters identified based on the expression of highly variable genes. Each dot depicts a single cell, colored according to cluster designation. Cell identities are annotated on the right. **(B)** Heat map showing the marker genes in each cell cluster identified through unsupervised clustering of kidney graft immune cells. Average expression scale is shown on the right. Cell identities are annotated as in **(A)**. **(C)** Bar plot showing the proportions of 20 immune cell populations in kidney allografts collected 7 days and 15 days following transplantation, respectively, colored according to cluster designation in **(A)**. UMAP, Uniform Manifold Approximation and Projection; Mφ, macrophage; DC, dendritic cell; NK, natural killer; pDC, Plasmacytoid dendritic cell.

Successful renal transplantation was modeled, and histological features of allografts used for scRNA-seq were confirmed by PAS staining after mice sacrificed on D7 (**Supplementary Figure 1B**). Regarding scRNA-seq data on D7, cell yield was 5,233 cells with the viability of 90%. Approximately 758 cells were filtered by quality control. Similarly 452 cells expressing markers of both T cells and macrophages/neutrophils were filtered as doublets. Next, 4,254 and 2,871 CD45^+^ leukocytes passing criteria of quality control were extracted from GSM4761000 and GSM4761003, respectively (**Supplementary Figures 2**, **3A–C**) (13). Then, Seurat was used for integrating D7 and D15 data, and unsupervised clustering (14). We identified 20 clusters of CD45^+^ cells and 1 cluster of erythroid cells, annotated them based on well-characterized marker genes (**Figures 2A, B**, **Supplementary Table 1**). The most abundant cell types were CD8^+^ T cells (clusters 0, 3, 4 and 8, Cd3e, Cd3g, Cd8a, Cd8b1) and monocytes/macrophages (clusters 1, 2, 6, 7, 12 and 16, Lyz2, Csf1r, Adgre1, Apoe, Aif1), constituting about 80% of the total leukocytes infiltrated (**Supplementary Table 1**). CD4^+^ T cells appeared in 2 distinct clusters (clusters 5 and 9, Cd3e, Cd3g, Cd4). We also identified 1 B cell cluster (cluster 10, Cd79a, Cd79b), 1 plasma cell cluster (cluster 19, Jchain, Iglv1, Iglc1), 1 neutrophil cluster (cluster 15, Csf3r, S100a8, S100a9, Ly6g, Lcn2), 1 natural killer (NK) cell cluster (cluster 13, Ncr1, Klrb1c, Gzma), 2 classical DC clusters (cDC; cluster 11, Flt3, Cd209a; cluster 17, Fscn1, Cacnb3, Mreg), 1 basophil cluster (cluster 18, Mcpt8, Gata2) and 1 plasmacytoid dendritic cell (pDC) cluster (cluster 20, Cox6a2, Klk1, Siglech). We then ordered these clusters according to cluster number in two timepoints separately and found a major reshuffling in cell populations (**Figure 2C**, **Supplementary Table 1**). The relative ratio of CD8 T cells dropped significantly from D7 to D15, mainly due to decreased proliferation and naïve CD8 T cell infiltration (**Figure 2C**, **Supplementary Table 1**). In addition, the proportion of both B cells and neutrophils decreased by about 3 folds (**Figure 2C**, **Supplementary Table 1**). On the contrary, the relative proportion of monocytes/macrophages and DCs increased significantly. Among them, Ly6c^lo^Mrc1+ macrophages increased by about 4.5 folds and a group of Ly6c^lo^Ear2+ macrophages increased by about 2.6 folds (**Figure 2C**, **Supplementary Table 1**)

### Monocytes/Macrophages

**Figure 3:**
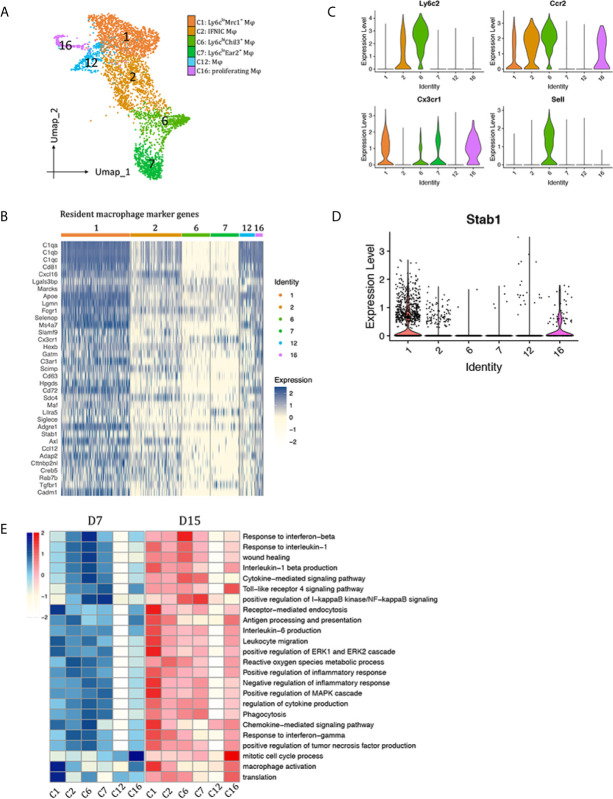
Macrophages subtypes presented in the allografted kidneys. **(A)** Integrated UMAP projection of the six macrophage clusters identified in the kidney grafts (clusters 1, 2, 6, 7, 12 and 16), colored according to cluster designation. Cell identities are annotated on the right. **(B)** Heatmap showing normalized expression of resident macrophage marker genes in macrophage clusters. Expression levels from low to high are colored from white to blue. Average expression scale is shown on the right. Violin plot showing normalized expression levels of the **(C)** Ccr2, Sell, Cx3cr1 and Ly6c2 **(D)** Stab1 in macrophage clusters. **(E)** Heatmap showing scaled GSVA score of selected GO terms. Average expression scale is shown on the left. UMAP, Uniform Manifold Approximation and Projection; D7, day 7; D15, day 15; Mφ, macrophage; GO, gene ontology; GSVA, gene set variation analysis.

We identified six distinct monocyte/macrophage clusters: clusters 1, 2, 6, 7, 12 and 16 (**Figure 3A**). Clusters 1, 12, 16 acquired a resident macrophage gene signature (**Figure 3B**) (15, 16). Other clusters were further divided into Ly6c^inter^ (cluster 2, Sell^-^Ccr2^+^Ly6c^inter^), Ly6c^hi^ (cluster 6, Ccr2^+^Sell^+^Cx3cr1^lo^Ly6c^hi^), and Ly6c^lo^ (cluster 7, Ccr2^-^Sell^-^Cx3cr1^hi^Ly6c^lo^) macrophages (**Figure 3C**).

To observe polarization of macrophage subtypes, expression of M1 and M2 markers were plotted in **Supplementary Figure 4**. Cluster 1 and 16 highly expressed M2 markers Mrc1, Clec10a and Selenop. Besides Mrc1, they also expressed another scavenger receptor Stab1 (**Figure 3D**), suggesting a role in scavenging debris/excess exracellular matrix. Ly6C^hi^ macrophages (cluster 6) significantly expressed M2 marker Chil3; Ly6C^inter^ macrophages (cluster 2) significantly expressed M1 markers Il1b and Nos2; Ly6C^lo^ macrophages (Cluster 7) also highly expressed Il1b; Tnf was widely expressed by all macrophage clusters, except cluster 12, a group of cells that do not express any of the above-mentioned polarization markers. Then, marker genes in each macrophage cluster were used for GO enrichment analysis (**Supplementary Figures 5A–F**). Top enriched biological pathways of each cluster were selected to compare the enrichment scores among macrophage subtypes by gene set variation analysis (GSVA, **Figure 3E**). In general, clusters 1, 2, 6, 7 contributed to inflammatory response during rejection, whereas cluster 12 seemed to be quite quiescent with no obvious functions. Cluster 16 is a group of proliferatin macrophages. Functionally, Ly6C^lo^Mrc1^+^ macrophages (cluster 1) may contribute to chemokine signaling pathway, receptor-mediated endocytosis, activation of ERK1 and ERK2 cascade. Ly6c^inter^Nos2^+^ macrophages (cluster 2) significantly correlated with response to IFNg, antigen processing and presentation, reactive oxygen species metabolic pathway, thus were annotated as IFNg induced cells (IFNIC). Ly6c^hi^Chil3^+^ macrophages (cluster 6) were enriched in pathways response to interferon beta and il1b, chemokine/cytokine signaling pathway, phagocytosis, and wound healing. Ly6C^lo^Ear2^+^ macrophages (Cluster 7) showed activation of I-kappaB kinase/NF-kappaB signaling pathway.

We next applied SCENIC analysis to assess which transcription factors (TFs) were responsible for the different gene profiles and functions among macrophage clusters (**Supplementary Figures 6A–H**) (11). SCENIC identified Maf, Tcfl2, Mitf and Nr1h3 as main candidate TFs underlying the specific gene expression in cluster 1 Ly6C^lo^Mrc1^+^ macrophages. Usf1, Spi1 and M1 polarization associated TFs Hif1a, stat1 were activated in IFNIC (cluster 2), whereas Cebpa, Xbp, Irf5 and Jund were activated in Ly6C^hi^Chil3^+^ macrophages (cluster 6). Cebpb, Etv6, Rel, Elf4, Elf2, Creb1, Elk3 and Smarcc2 were underlying the specific gene expression in Ly6C^lo^Ear2^+^ macrophages (cluster 7). TFs related to proliferation, including E2f2, E2f7, E2f8 and Mybl2, were specifically activated in cluster 16.

### Monocle Pseudotime Analysis Revealed Potential Paths of Infiltrating Monocytes/Macrophages Differentiation During Kidney Allograft

**Figure 4:**
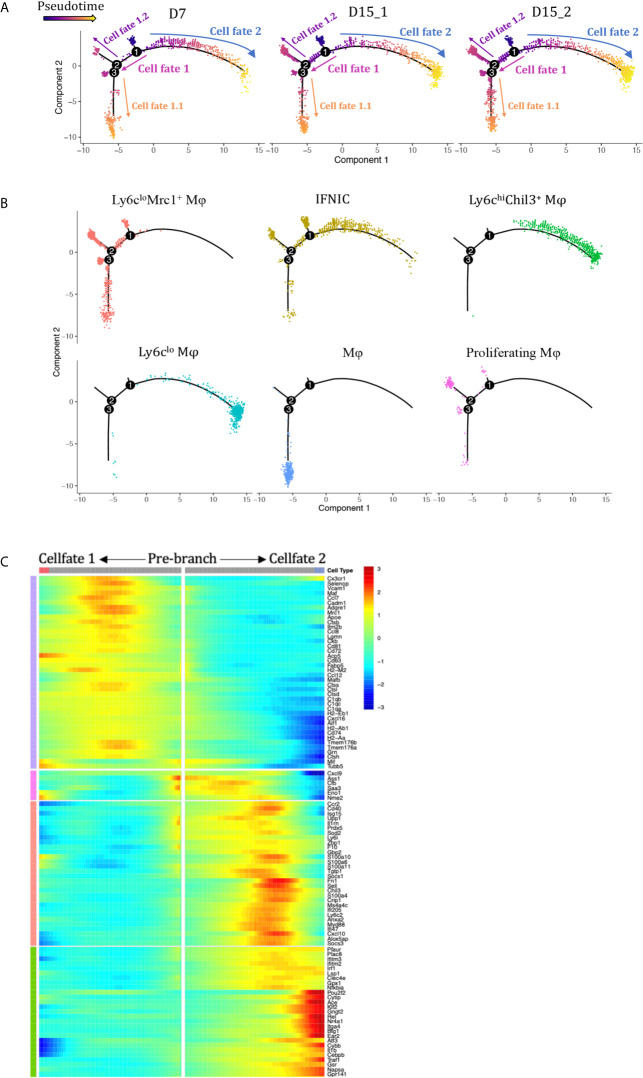
Analysis of macrophage differentiation trajectories in the transplanted kidney. Pseudotime analysis in Monocle split according to **(A)** rejection types and **(B)** seurat clusters. Each dot represents a single cell. **(C)** Heatmap of pseudotime gene expression variation on branches of the pseudotime tree (as indicated in **A**, only top 106 variable genes are shown). D7, day 7; D15, day 15; Mφ, macrophage; IFNIC, IFNg induced cells.

We performed pseudotime analysis to infer possible monocytes/macrophages developmental trajectories, with a clear continuum from state IFNIC monocytes/macrophages and Ly6C^hi^ macrophages to Ly6C^lo^Ear2^+^ macrophages (cell fate 2, **Figures 4A, B** **)** or Ly6C^lo^Mrc1^+^ macrophages (cell fate 1, **Figures 4A, B** **)**. Clusters corresponding to non-functional macrophages (cluster12, cell fate 1.1, **Figure 4A**) and proliferating macrophages (cluster12, cell fate 1.2, **Figure 4A**) were placed at two ends after a trajectory bifurcation 2. IFNg-induced proteins (Cxcl9, Cxcl10, Saa3, Ifitm2, Ifitm3, Socs1) occupied specific pre-branch of pseudotime-dependent genes heatmap, which clustered genes following similar kinetic trends (**Figure 4C**, **Supplementary Table 2**). Corresponding to the trajectory bifurcation 1, cells were on the one hand led to one fate highly expressing a set of transcripts including Nr4a1, Ear2, Cebpb, Klf2, Pouf2f, Trem3, Gngt2, Il1b and Ace etc. (Cell Fate 2, **Figure 4C**) while on the other hand following the opposite trajectory and acquiring expression of MHCII encoding genes, scavenger receptor gene Mrc1 and resident macrophage markers Cd81, Mafb, Maf and Cxcl16 etc. (Cell Fate 1, **Figure 4C**).

### Blunted Response to Interferon Gamma Contributed to AR Chronicization

**Figure 5:**
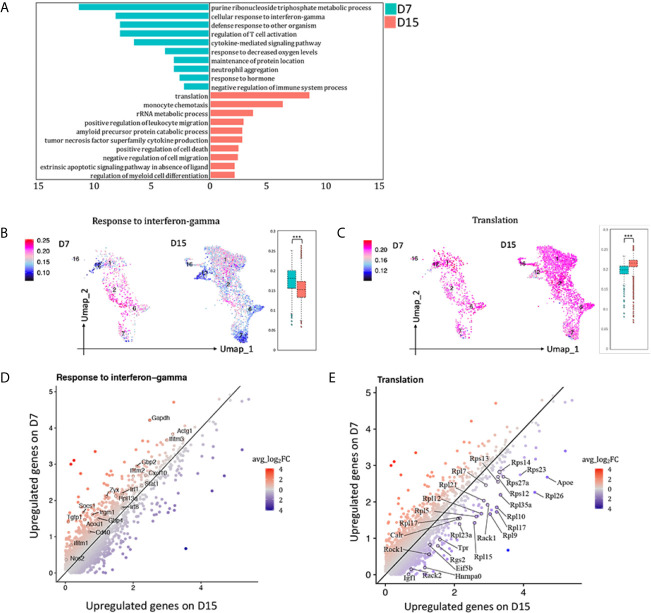
Blunted response to interferon gamma contributed to acute rejection chronicization. **(A)** Differences in hallmark pathways of macrophages from different rejection types as determined by Metascape. Feature plot and bar plot showing gene-set activity scores of GO pathway **(B)** response to interferon gamma, **(C)** translation. Each point depicts a single cell, colored according to normalized gene-set activity scores. Average scale is shown on the left. Scatter plot depicting DEGs enriched in pathway **(D)** response to interferon gamma and **(E)** translation. Each dot represents a gene, colored according to the avg_log_2_FC from white to blue or red. Only DEGs with avg_log_2_FC >0.59 were circled and labeled. D7, day 7; D15, day 15; avg_log_2_FC, average log_2_ fold change; adj_p value, adjusted p value. ***p < 0.001.

To observe the functional changes of macrophages from AR towards CR, differential expression genes (DEGs) comparing total macrophages (cluster 1, 2, 6, 7, 12, 16) on D7 versus D15 were calculated. 292 DEGs with avg_ log_2_FC >0.59, adj_p value <0.01 were upregulated on D7 whereas 197 were upregulated on D15 (**Supplementary Figure 7A**). GO enrichment analysis results were shown in **Figure 5A**. In the meanwhile, GSVA analysis was conducted (**Supplementary Figure 7B**). Both indicated that macrophages infiltrated in kidney allografts on D7 following transplantation were more activated in the pathway response to IFNg than those on D15 (**Figures 5B, D**; **Supplementary Figure 7B**). Representative DEGs enriched in response to IFNg (Nos2, Socs1, Stat1, Cxcl10, Mif, etcetera) were marked in **Figure 5D**. Interestingly, we found a significant number of ribosomal protein genes that were highly and consistently expressed on D15 than D7 (**Figures 5C, E** **)**, resembling a tolerized gene signature reported by others (13).

### CD8 T Cells

**Figure 6:**
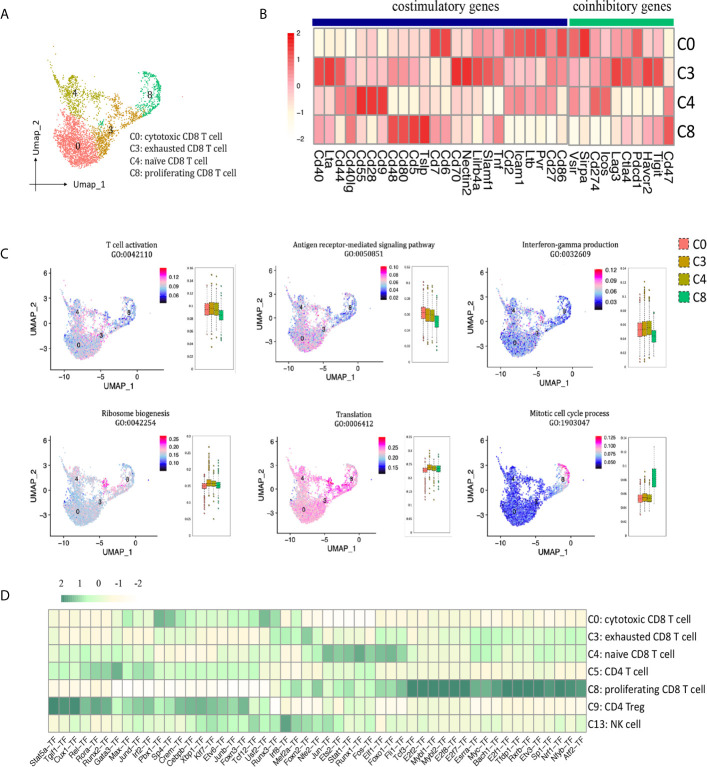
CD8 T cell presented in the allografted kidneys. **(A)** Integrated UMAP projection of the four CD8 T cell clusters identified in the kidney grafts (clusters 0, 3, 4 and 8), colored according to cluster designation. Cell identities are annotated on the right. **(B)** Heatmap showing costimulatory and coinhibitory genes in CD8 T cell clusters. Scale bar is shown on the left. **(C)** Feature plots showing AUC scores of GO pathways in CD8 T cell. Cell identities are annotated above the cell cluster. Score bar is shown on the right. **(D)** Heatmap of the area under the curve scores for the expression of gene sets regulated by transcription factors, as estimated with SCENIC, in CD8 T cell, CD4 T cell and NK cell clusters. Scaled AUC score bar is shown above. UMAP, Uniform Manifold Approximation and Projection; AUC, area under the curve; SCENIC, single-cell regulatory network inference and clustering; NK, natural killer.

In allograft rejection, infiltrating CD8 T cells play a major role in tubulitis and tissue necrosis (17, 18). We identified 4 clusters of CD8 T cells (**Figures 2B**, **6A**): cytotoxic CD8 T cells (cluster 0, Nkg7^+^, Gzmb^+^, Fasl^+^, Ifng^+^, Itgae^+^), exhausted CD8 T cells (cluster 3, Pdcd1^+^, Lag3^+^, Havcr2^+^, Tigit^+^), naïve CD8 T cells (cluster 4, Il7r^+^, Tcf7^+^, Lef1^+^), proliferating CD8 T cells (cluster 8, Mki67^+^, Top2a^+^). Cytotoxic CD8 T cells (C0) highly expressed costimulatory genes Pvr, Ltb, Cd6, Cd7, Icam1, and coinhibitory genes Pdcd1 and Sirpa; exhausted CD8 T cells (C3) expressed the richest coinhibitory genes Tigit, Havcr2, Ctla4, Lag3 and Pdcd1; naïve CD8 T cells (C4) highly expressed costimulatory gene Cd28 (**Figure 6B**). GO enrichment analysis showed that clusters 0, 3 and 4 were enriched in T cell activation, antigen receptor-mediated signaling pathway and IFNg production (**Figure 6C**). Naïve CD8 T cells (cluster 4) were involved in translation and ribosome biogenesis. Proliferating CD8 T cells (cluster 8) gained high score in mitotic cell cycle process. SCENIC suggested Pbx1, Sp4, Usf2 and Runx3 as main candidate TFs underlying the specific gene expression in cytotoxic CD8 T cells (**Figure 6D**, cluster 0). Genes regulated by TFs Runx1, Foxo1, Ets2, Elf1 and Jun were also upregulated in naïve CD8 T cells (**Figure 6D**, cluster 4). Genes regulated by TFs Tfdp1, Mybl2, E2f8, E2f7 and E2f2, etc. were specific to proliferating CD8 T cells (**Figure 6D**, cluster 8).

### CD4 T Cells

**Figure 7:**
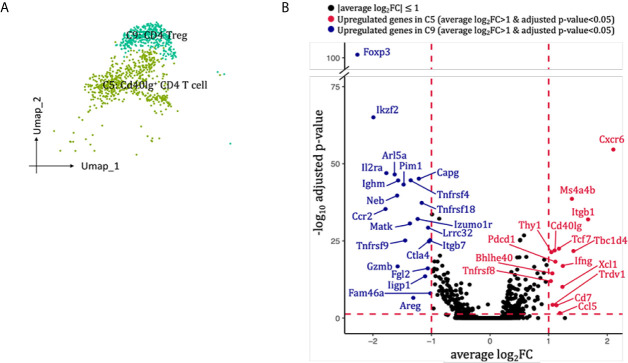
CD4 T cells presented in the allografted kidneys. **(A)** Integrated UMAP projection of the two CD4 T cells clusters identified in the kidney grafts (clusters 5 and 9), colored according to cluster designation. Cell identities are annotated on the clusters. **(B)** Volcano plots of the differentially modulated genes in C5 versus C9. The x axis specifies the average log_2_FC and the y axis specifies the negative logarithm (base 10) of the adj_p value. Red vertical and horizontal lines reflect the filtering criteria (avg_log_2_FC = ± 0.59 and adj_p value = 0.05). Red dots indicate genes induced in C5; blue dots indicate genes induced in C9. Genes passing filtering criteria are labeled. UMAP, Uniform Manifold Approximation and Projection; avg_log_2_FC, average log_2_ fold change; adj_p value, adjusted p value.

Two clusters of CD4 T cells were identified in our scRNA-seq data (**Figures 2B**, **7A**). Cluster 5 (Cd40Ig^+^CD4 T cell) highly expressed Cxcr6, Ccl5, Ifng and co-stimulatory marker Cd40Ig, whereas cluster 9 (Treg cell) expressed mRNA signals for key Treg markers Foxp3, Il2ra, Tnfrsf18, Ctla4 and Il10 (**Figure 7B**). Neither of the two clusters expressed classical T helper cell 2 markers Il4 and Il13, or T helper cell 17 marker Il17a (**Supplementary Figure 8A**). When we re-clustered all the into 4 CD4 T cell clusters, three clusters (0, 2, 3) expressed Th1 marker Ifng but none expressed significant Th2 or Th17 marker genes (**Supplementary Figure 8A-D**). Thus, we failed to dissect T helper subtypes even at higher resolution, possibly due to insufficient number of CD4 T cells sequenced. SCENIC identified Gata3 as main candidate TF underlying the specific gene expression in Cd40Ig^+^CD4 T cell (**Figure 6D**, cluster 5), Stat5a, Tgif1, Cux1 and Runx2 as main candidate TF underlying the specific gene expression in CD4 Treg (**Figure 6D**, cluster 9).

### NK Cells

**Figure 8:**
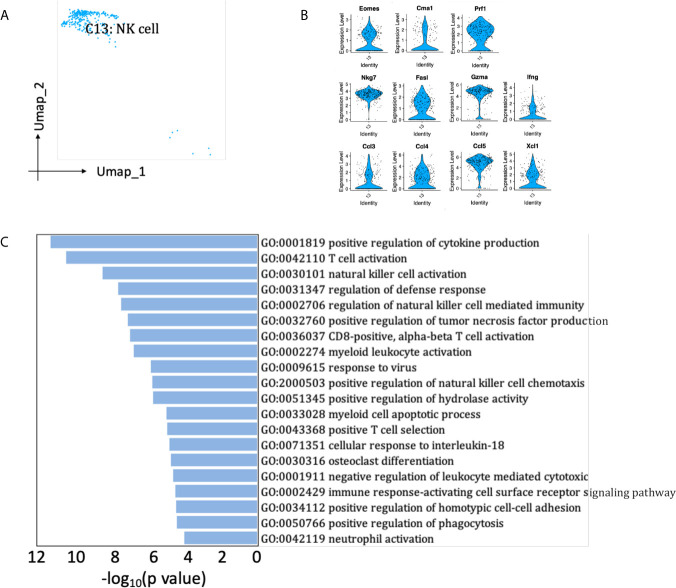
NK cells presented in the allografted kidneys. **(A)** Integrated UMAP projection of the NK cell cluster identified in the kidney grafts (clusters 13), colored according to cluster designation. Cell identity is annotated on the cluster. **(B)** Violin plot showing normalized expression levels of markers in NK cell cluster. **(C)** Pathway enrichment of genes with avg_log_2_FC ≥0.59 in NK cell cluster. UMAP, Uniform Manifold Approximation and Projection; avg_log_2_FC, average log_2_ fold change.

A small population of renal allograft NK cells were identified by NK-specific markers (**Figures 2B**, **8A**), expressing Eomes, a T-box transcription factor expressed by activated CD8 T cells and NK cells (**Figure 8B**) (19). They also significantly expressed mRNA for proinflammatory cytokine Ifng and chemokine Ccl3 (MIP-1α), Ccl4 (MIP-1β), Ccl5, Xcl1, which attract effector lymphocytes and myeloid cells toward inflamed tissues (**Figure 8B**) (20). Besides, NK cells expressed cytotoxic marker genes Prf1, Gzma, Fasl, playing roles in the regulation of natural killer cell mediated cytotoxicity and positive regulation of cytokine production (**Figure 8C**). SCENIC identified Irf8 as main candidate TF underlying the specific gene expression in NK cells (**Figure 6D**, cluster 13), which regulated natural killer cell development (21).

### B Cells

**Figure 9:**
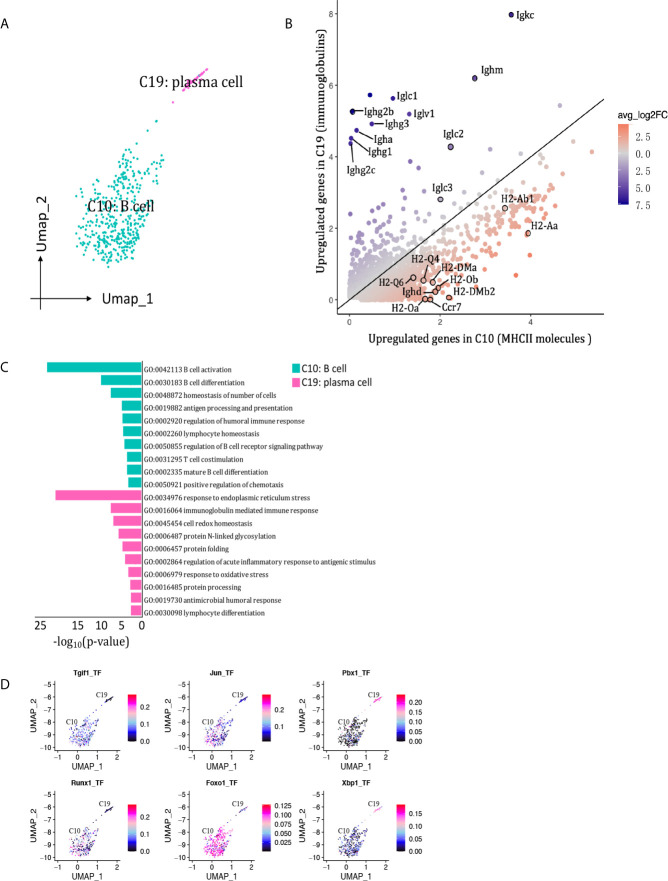
B cells presented in the allografted kidneys. **(A)** Integrated UMAP projection of the 2 B cell clusters identified in the kidney grafts (clusters 10 and 19), colored according to cluster designation. Cell identities are annotated on the clusters. **(B)** Scatter plot showing average expression of genes in C5 versus C9. Each dot represents a gene, colored according to the average log_2_FC from white to blue or red. Genes encoding immunoglobulins and MHCII molecules with |avg_log_2_FC| ≥0.59 were circle and labeled. **(C)** Pathway enrichment of genes with avg_log_2_FC ≥0.59 in clusters 10 and 19. **(D)** Feature plot showing AUC scores for the expression of gene sets regulated by transcription factors, as estimated with SCENIC, in clusters 10 and 19. UMAP, Uniform Manifold Approximation and Projection; avg_log_2_FC, average log_2_ fold change; adj_p value, adjusted p value; AUC, area under the curve; SCENIC, single-cell regulatory network inference and clustering.

We detected 469 B cells that formed two clusters (**Figure 9A**), with the proportion dropping from 7.85% on D7 to 1.38% on D15 (**Figure 2C**, **Supplementary Table 1**). Cluster 10 was the major B cell population at both peak and regression phases, characterized by Cd79a, Cd79b and Siglecg (**Figure 2B**). Cluster 19 (plasma B cell) was marked by Jchain and Eaf2. Besides, cluster 10 expressed MHCII molecules and Ighd at higher levels, whereas cluster 19 (plasma B cells) highly expressed genes encoding immunoglobulins, including Ighg2b, Igha, Iglc1, Iglc2, Iglv2, and Ighj3, etcetera (**Figure 9B**). The functions of B cell cluster 10 included translation, antigen processing and presentation of peptide antigen *via* MHC class II, response to interferon-gamma. The functions of plasma cells (cluster 19) included response to endoplasmic reticulum stress and immunoglobulin production (**Figure 9C**). SCENIC identified Foxo1, Runx1, Jun and Tgif1 as main candidate TFs underlying the specific gene expression in B cell cluster 10, Pbx1 and Xbp1 as main candidate TFs underlying the specific gene expression in plasma cell cluster 19 (**Figure 9D**).

### Granulocytes

**Figure 10:**
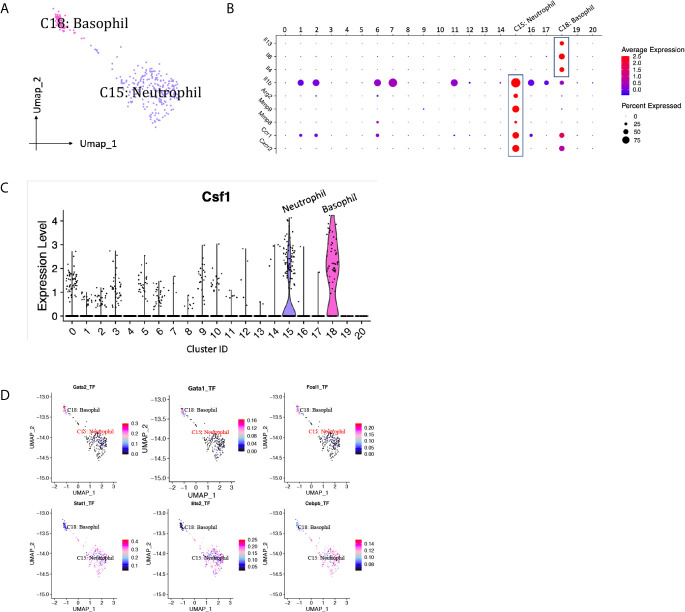
Granulocytes presented in the allografted kidneys. **(A)** Integrated UMAP projection of one neutrophil cluster (cluster 15) and one basophil cluster (cluster 18) identified in the kidney grafts, colored according to cluster designation. Cell identities are annotated above the cell dots. **(B)** Dot plot showing gene expression levels of Il13, Il4, Il6, Il1b, Arg2, Mmp8, Mmp9, Ccr1 and Cxcr2 in all clusters. **(C)** Violin plot showing normalized expression levels of Csf1 in all clusters. **(D)** Feature plot showing the AUC scores for the expression of gene sets regulated by transcription factors, as estimated with SCENIC, in neutrophil and basophil cluster. AUC score bar is shown on the right. UMAP, Uniform Manifold Approximation and Projection; AUC, area under the curve; SCENIC, single-cell regulatory network inference and clustering.

Neutrophils are usually the first leukocytes to infiltrate into transplanted organs and are a well-established marker of transplant injury (22). Only one neutrophil cluster (cluster 15) with 228 cells was identified in our scRNA-seq data, constituting <5% of the immune spectrum (**Figure 10A**, **Supplementary Table 1**). The proportion of neutrophils dropped about 3.5 folds from D7 to D15 (**Supplementary Table 1**). Featured higher expression of mRNA for pro-repair genes Mmp8, Mmp9 and Arg2 were detected in neutrophil cluster, resembling a cluster of pro-repair subset reported by others (**Figure 10B**) (6). Besides, Il1b was expressed at particular high levels in neutrophils, contributing to the pathogenesis of immune response during inflammation. Basophils were also present in the majority of renal biopsies showing T cell-mediated rejection and were absent in biopsies without rejection (23). A small population of basophils (cluster 18, <1%) was identified by expressing basophil marker Mcpt8, Gata2 (**Figure 2B**) and activation markers, including type 2-associated cytokines Il4, Il13, and proinflammatory marker Il6 (**Figure 10B**, **Supplementary Table 1**). CSF1, a critical growth factor for macrophage development, was predominantly expressed by granulocytes, including neutrophils and basophils, among all the immune cells (**Figure 10C**). SCENIC identified Stat1, Ets2 and Cebpb as main candidate TFs underlying the specific gene expression in neutrophils, Gata1, Gata2 and Fosl1 as main candidate TFs underlying the specific gene expression in basophils (**Figure 10D**).

### DCs

**Figure 11:**
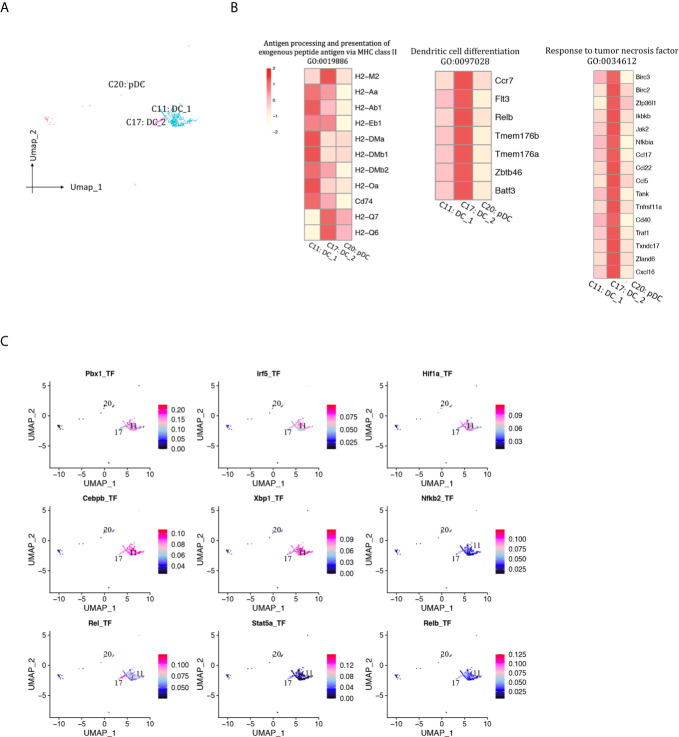
DCs presented in the allografted kidneys. **(A)** Integrated UMAP projection of three DC cell clusters (11, 17, 20) identified in the kidney grafts, colored according to cluster designation. Cell identities are annotated above the cell dots. **(B)** Heatmap showing scaled expression of genes enriched in GO pathways. Scale bar is shown on the left top. **(C)** Feature plot showing the AUC scores for the expression of gene sets regulated by transcription factors, as estimated with SCENIC, in clusters 11 and 17. AUC score bar is shown on the right. DC, dendritic cell; pDC, plasmacytoid dendritic cell; UMAP, Uniform Manifold Approximation and Projection; GO, gene ontology; AUC, area under the curve; SCENIC, single-cell regulatory network inference and clustering.

A total of 469 DCs were detected and separated into two cDC clusters and one pDC cluster (**Figures 2B**, **11A**, **Supplementary Table 1**). DC1 (Cd209a^+^Flt3^+^, cluster 11) was the major DC population at both phases. The proportion of both DC1 and DC2 (Fscn1^+^Cacnb3^+^Mreg^+^, cluster 17) increased as rejection regressed, whereas pDC (Siglech^+^Cox6a2^+^Ccr9^+^, cluster 20) proportion was at low levels throughout this process (<0.5%). DC1 expressed higher levels of MHCII molecules than DC2 (**Figure 11B**). DC2 was associated with response to tumor necrosis factor and DC differentiation. TFs including Pbx1, Irf5, Hif1a and Cebpb were underlying the specific gene expression in DC1, whereas Rel, Relb, Nfkb2 and Stat5a were underlying the specific gene expression in DC2 (**Figure 11C**).

### Complex Intercellular Communication Networks Among Immune Cells

**Figure 12:**
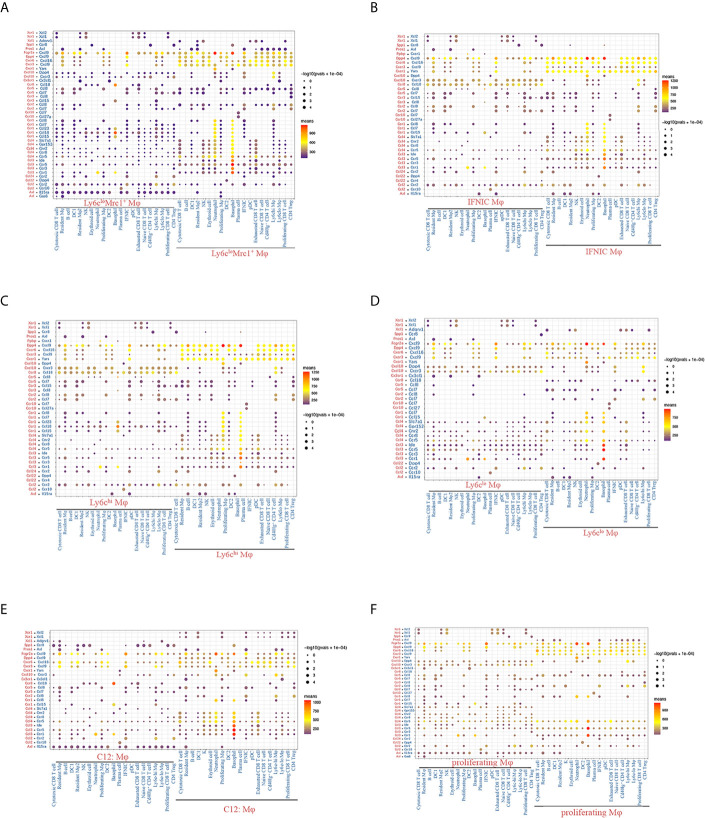
Chemokine mediated LR interactions between macrophages and other immune cell clusters. **(A–F)** Overview of chemokine mediated LR interactions between macrophages and other immune cell clusters. P values are indicated by circle size, with the scale to the right (permutation test). The means of the average expression levels of interacting molecule 1 in cluster 1 and interacting molecule 2 in cluster 2 are indicated by color. Assays were carried out at the mRNA level but were used to extrapolate protein interactions. Only LR pairs with means value >100 are shown. Mφ, macrophage; DC, dendritic cells; pDC, plasmacytoid dendritic cell; IFNIC, IFNg induced cells; LR, ligand and receptor.

Next, we used CellPhoneDB to identify LR pairs and molecular interactions among the major cell types (12, 24). Broadcast ligands for which cognate receptors were detected demonstrated extensive communications among immune cells (**Supplementary Figures 9A, B**). Among them, myeloid cells harbored the highest pair numbers, indicating a wide range of cell-to-cell interactions between myeloid cells and other cells. Highest chemokine mediated LR interactions were detected in granulocytes (**Supplementary Figures 10A, B**), followed by macrophages (**Figures 12A** **–F**). Notably, both macrophages and cDCs commonly expressed relatively high levels of chemokines (e.g., Cxcl9, Cxcl16, Yars and Cxcl10), whereas, the corresponding receptors were widely expressed in other immune cells, suggesting that these chemokines play significant roles in interactions within myeloid cells or between myeloid cells and other cells (**Figures 12A–F**, **Supplementary Figures 11A, B**). Compared to other macrophage clusters, Ly6c^lo^Ear2^+^ macrophages exhibited significantly less and low means of LR pairs, lacking expression of receptors including Ccr1, Ccr2, Ccr5 and Ccr8 (**Figure 12D**). Ly6c^lo^Mrc1^+^ macrophages (cluster 1, 16) and IFNIC (cluster 2) specifically expressed ligand Axl and interacted with other cells through Axl-Il15ra, Axl-Pros1 pairs (**Figures 12A, C** **, F**, **Supplementary Figure 11C**). It was reported that Axl augmented intragraft differentiation of certain types of proinflammatory macrophages and enhanced early allograft inflammation (13).

## Discussion

Here, we presented, for the first time to our knowledge, a single-cell resolution catalog of immune cells profiling from AR towards CR during mouse renal allograft rejection. It is reasonable to integrate our single-cell data of D7 with D15 data extracted from GSE157292, as kidney transplant models were both built by transplanting kidneys from BALB/c into B6 mice. Though Dangi et al. bilaterally nephrectomized the native kidneys while we left them in situ, the histological outcomes on D7 are comparable between two mouse models (8). In addition, two scRNA-seq studies were both performed on 10× genomics platform. There were several novel findings from this study.

First, we found a significant change in the proportion of immune cells in the grafted kidneys between AR and mix rejection. Generally, the proportion of macrophages increased from 21.11 to about 50%, including Ly6C^lo^Mrc1^+^ macrophages (clusters 1, 16) and Ly6C^lo^Ear2^+^ macrophages (cluster 7). Pseudotime analysis indicated that both Ly6C^lo^Mrc1^+^ and Ly6C^lo^Ear2^+^ macrophages were at the terminal differentiation stage. To the opposite, CD8 T cells dropped from 51.44 to about 28%, mainly due to the decrease in proliferating CD8 T cells (cluster 8) and naïve CD8 T cells (cluster 4). In addition, B cells and neutrophils also showed significant decrease.

Second, the macrophage polarization with distinctive functions may contribute to renal allograft progression or regression. Ly6C^hi^ monocytes recruitment could be induced by type I IFN signaling (25, 26). During acute renal ischemic-reperfusion injury, it was found that the CD11b^+^Ly6C^hi^ macrophage population was associated with the onset of renal injury and increased expression of proinflammatory cytokines, whereas the Ly6C^inter^ population had a distinct phenotype of wound healing and the Ly6C^lo^ population exhibited a profibrotic phenotype as previously reported (27). These findings indicate that the macrophage phenotypes may contribute significantly to the progression or regression of renal allograft outcomes. Interestingly, by GO enrichment and GSVA analysis, we also showed that Ly6C^hi^Chil3^+^ (coding Ym1) macrophages (cluster 6) responding to type I IFN and Il1b were enriched in wound healing and phagocytosis, contributing to the resolution of inflammation and tissue repair. Ly6C^lo^ macrophages could be further clustered into Ly6C^lo^Ear2^+^ macrophages (Cluster 7) with weak chemoattraction and Ly6C^lo^Mrc1^+^ macrophages (Clusters 1 and 16) acquiring a resident macrophage-like gene signature, both increased in the process towards CR. IFNIC (cluster 2), induced by IFNg and ROS, also gained high GSVA score in antigen processing and presentation. Pseudotime trajectories indicated that IFNIC may be a heterogeneous subtype. These findings suggested that although Ly6C is used to classify macrophages, functions of macrophage subtypes could be altered during the renal allograft progression or regression. This can also be observed in other disease models. For example, in unilateral ureteral obstruction model, kidney infiltrating immature pro-inflammatory Ly6C^hi^ macrophages can progressively differentiate into mature profibrotic or M2 Ly6C^lo^ macrophages (28, 29). In mouse cardiac transplantation, recipient derived suppressive Ly6C^lo^ macrophages show inhibitory effect on CD8^+^ T cell immunity while promoting Treg cell expansion. Besides, immunogenic Ly6C^hi^ macrophages could develop into tolerogenic Ly6C^lo^ macrophages under the action of neutrophil derived CSF1 (30, 31). Despite the generally pro-inflammatory effects of Ly6C^hi^ monocytes/macrophages, it has also been reported that a cluster of bone marrow originated Ym1^+^Ly6C^hi^ monocytes exhibited immunoregulatory and tissue-reparative phenotypes during the recovery phase of colitis (32). In addition, Ly6C^lo^ macrophages may also be pro-inflammatory and induce kidney injury in acute kidney injury mice (33). Thus, macrophages are highly heterogeneous and participate in the disease inflammation or resolution.

Third, the present study proved IFNg as an important inducer of macrophage and cDC activation during mouse renal allograft rejection. T lymphocytes and NK cells significantly expressed IFNg (**Figure 2B**). Macrophage subtypes and cDC clusters were also enriched in pathway response to IFNg (**Supplementary Figures 6A–H**). Importantly, both DEGs enrichment and GSVA analysis also showed that IFNg response decreased from the inflammation peak on D7 to D15. In addition, we found a wide range of LR pairs among immune cells. Granulocytes and macrophages interacted most extensively with other cell types. Ligation by IFNg induced chemokines like Cxcl9, Cxcl16 and Cxcl10 in macrophages and cDC mediated the activation of other cells. Indeed, IFNg is needed for transplant rejection by inducing MHC products and pro-inflammatory molecules in both human and mouse kidneys (34–39). *In vitro* study also proves that IFNg can stimulate the maturation of DC by upregulating MHCII molecules and costimulatory molecules CD40 and CD80 (40). Thus, decrease in IFNg response by renal macrophages and cDCs may contribute to inflammation regression during renal allograft regrection.

So far, only limited information on the use of scRNA-seq in murine kidney transplantation has been published. Anil Dangi et al. reported whole transcriptomics of mouse kidney transplants on day 15 in a single cell level and identified 13 immune cell clusters (13). In the present study, we developed further in-depth analysis of these immune cell types with comprehensive classification and description of cell subtypes including their specific marker genes, functional characteristics, transcriptional regulation, and the proportional dynamics from the renal allograft rejection peak on D7 to the regression phase on D15. In naïve mouse kidneys, monocytes/macrophages, neutrophils, DCs, B and T lymphocytes, NK cells could all be observed (16, 41, 42). Among macrophage subtypes, infiltrating Ly6C^hi^Chil3^+^ macrophage and Ly6C^lo^ patrolling macrophages are commonly found in normal kidneys (16, 42), whereas IFNIC and Mrc1^+^ macrophages are more common to disease models like unilateral ureteric obstruction model (42). Study of kidney allograft biopsies from human recipients undergoing AR and CR also identified immune populations like those in mice, including monocytes, B cells, T cells, plasma cells (43–45), therefore independently supporting a role of these cells in kidney rejection in humans. However, due to the limited size of human biopsy samples and the large percentage of parenchymal cells, further analysis of immune cell subtypes is lacking. Thus, in-depth analysis of immune cells in human kidney biopsies is needed. In summary, results from this study may provide more comprehensive insight for our better understanding of the immune cell profiling from AR towards CR and may also provide valuable information for the future studies in immunological targeting and tolerance.

## Data Availability Statement

Raw scRNA-seq data (fastq files) and processed unique molecular identifiers count matrices used in the current study are publicly available on NCBI GEO (GSE166775).

## Ethics Statement

The animal study was reviewed and approved by Animal Experimentation Ethics Committee at the Chinese University of Hong Kong.

## Author Contributions

QS, JHC, HJ, and HL conceptualized the study. QS, YW, JYC, and XH performed experiments. QS and LM analyzed bioinformatics. JYC, HJ, HL, and ST provided reagents and animals. QS drafted the manuscript. JYC, HJ, and HL edited and finalized the paper. All authors contributed to the article and approved the submitted version.

## Funding

This research was supported by National Natural Science Foundation of China: B3436, B1903, 81170697, 91770752, 81970651; Research Grants Council of Hong Kong (CRF 14104019, 14117418, 14121816, and C7018-16G); the Lui Che Woo Institute of Innovative Medicine (CARE program), and Guangdong-Hong Kong-Macao-Joint Labs Program from Guangdong Science and Technology Department (2019B121205005).

## Conflict of Interest

The authors declare that the research was conducted in the absence of any commercial or financial relationships that could be construed as a potential conflict of interest.

## Abbreviation

scRNA-seq, single cell RNA sequencing; D7, day 7; D15, day 15; AR, acute rejection; CR, chronic rejection; DCs, dendritic cells; LR, ligand and receptor; IFNg, interferon gamma; MHC, major histocompatibility complex; HE, hematoxylin & eosin;PAS, periodic acid–Schiff;ELISA, Serum Enzyme-Linked ImmunoSorbent Assay; FACS, fluorescence-activated cell sorting; GEO, Gene Expression Omnibus; UMAP, Uniform Manifold Approximation and Projection; DEGs, differential expression genes; GO, Gene Ontology; GSVA, gene set variation analysis; adj_p value, adjusted p value; SCENIC, single-cell regulatory network inference and clustering; TFs, transcription factors; NK, natural killer; cDC, classical dendritic cells; pDC, plasmacytoid dendritic cell; IFNIC, IFNg induced cells.
