# Supplementary material for: Single-Cell RNA Sequencing Reveals the Immunological Profiles of Renal Allograft Rejection in Mice
Source: Front Immunol. 2021 Jul 22;12:693608. doi: 10.3389/fimmu.2021.693608 (PMC8340022; doi:10.3389/fimmu.2021.693608)
Supplement: Supplementary file 1 [file DataSheet_1.pdf]

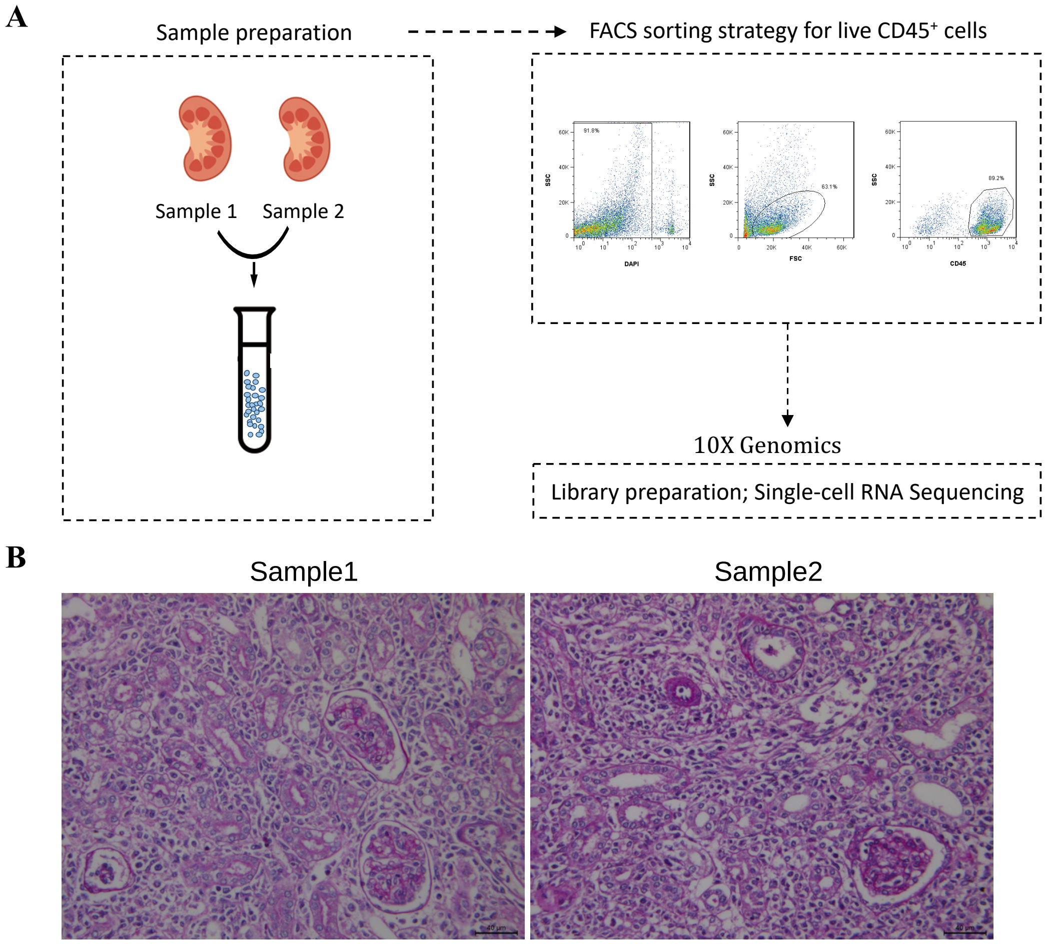

Supplementary Figure 1. (A) Graphical representation of the scRNA sequencing experimental setup. Samples were isolated from kidney grafts of recipient mice 7 days after surgery. Two samples were pooled. **(B)** Representative photomicrographs showing histopathology (by PAS staining) of the kidneys harvested from recipient mice used for scRNA-seq analysis. Scale bar: 40 µm.

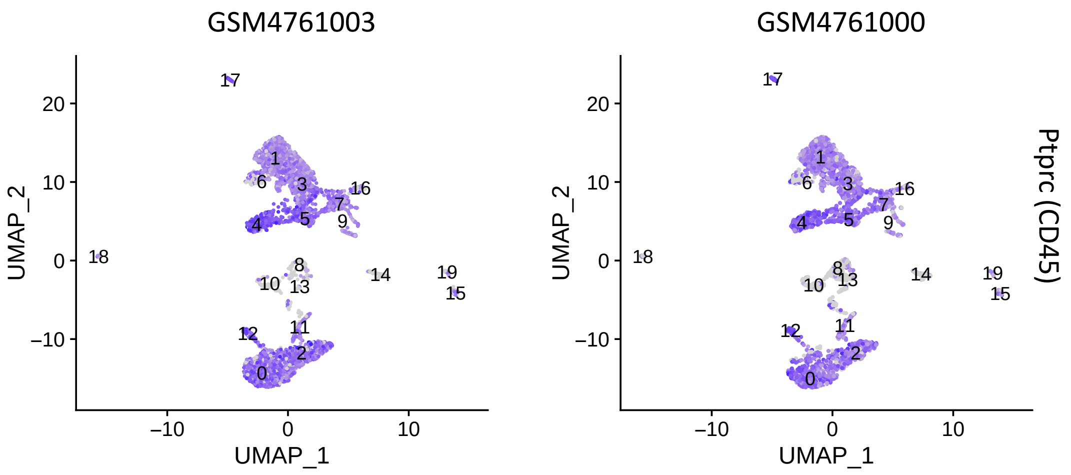

Supplementary Figure 2. Feature plot of Ptprc (coding CD45) expression level in cell clusters identified in data set GSM4761003 and GSM4761000. Cluster 0:7, 9, 11, 12, 15:19 were extracted as leukocytes.

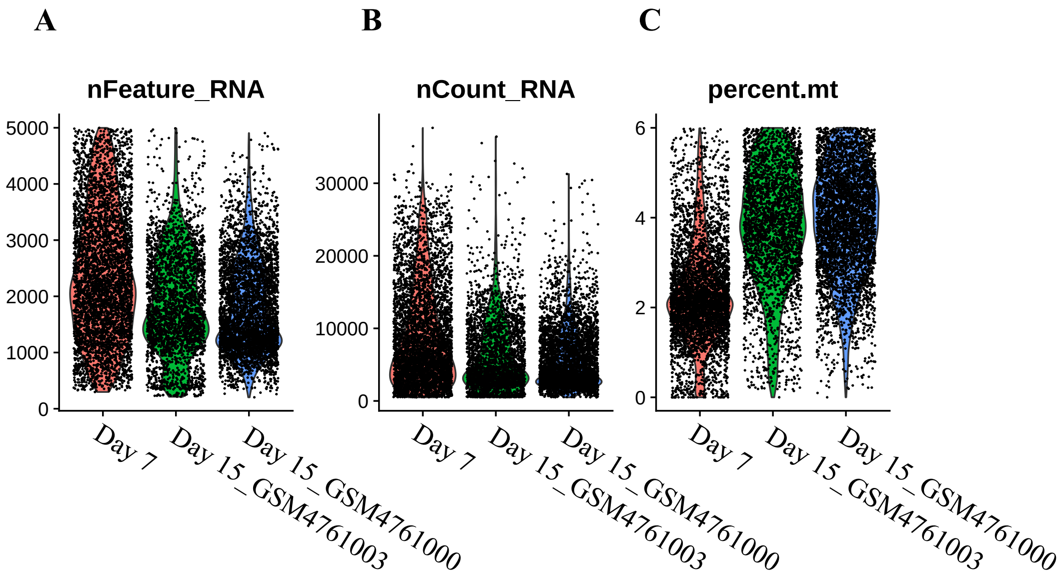

Supplementary Figure 3. Violin plot showing quality control results. The number of genes detected in each cell **(A)**, the total number of molecules detected within a cell **(B)** and the percentage of mitochondrial gene detected in each cell **(C)** after quality control.

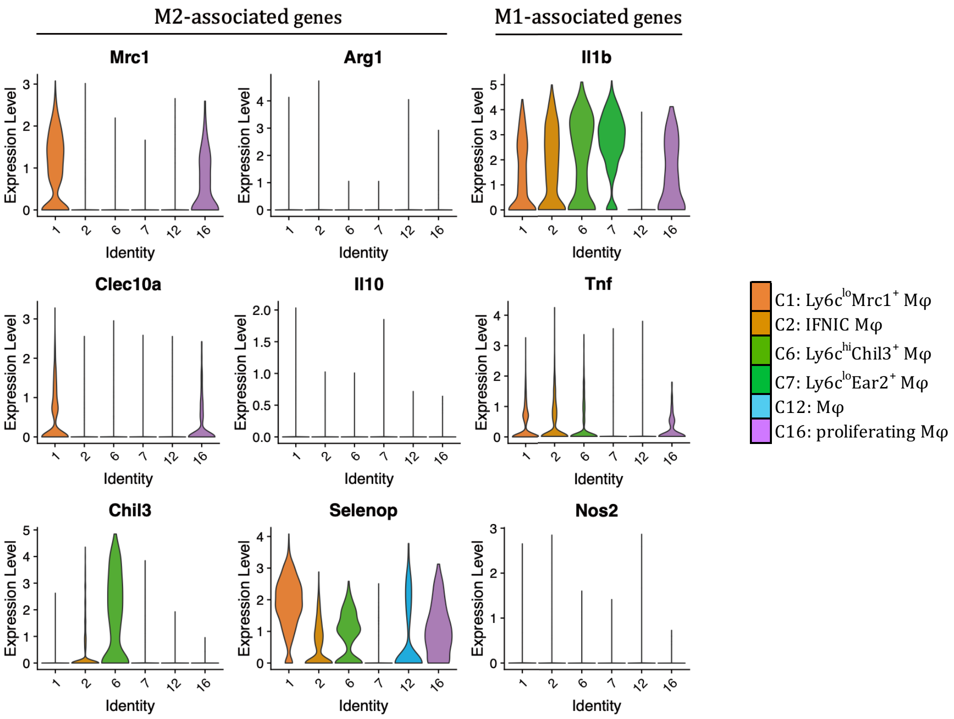

Supplementary Figure 4. Violin plot showing normalized expression levels of the M1 and M2 markers in macrophage subtypes. Cell identities are annotated on the right.

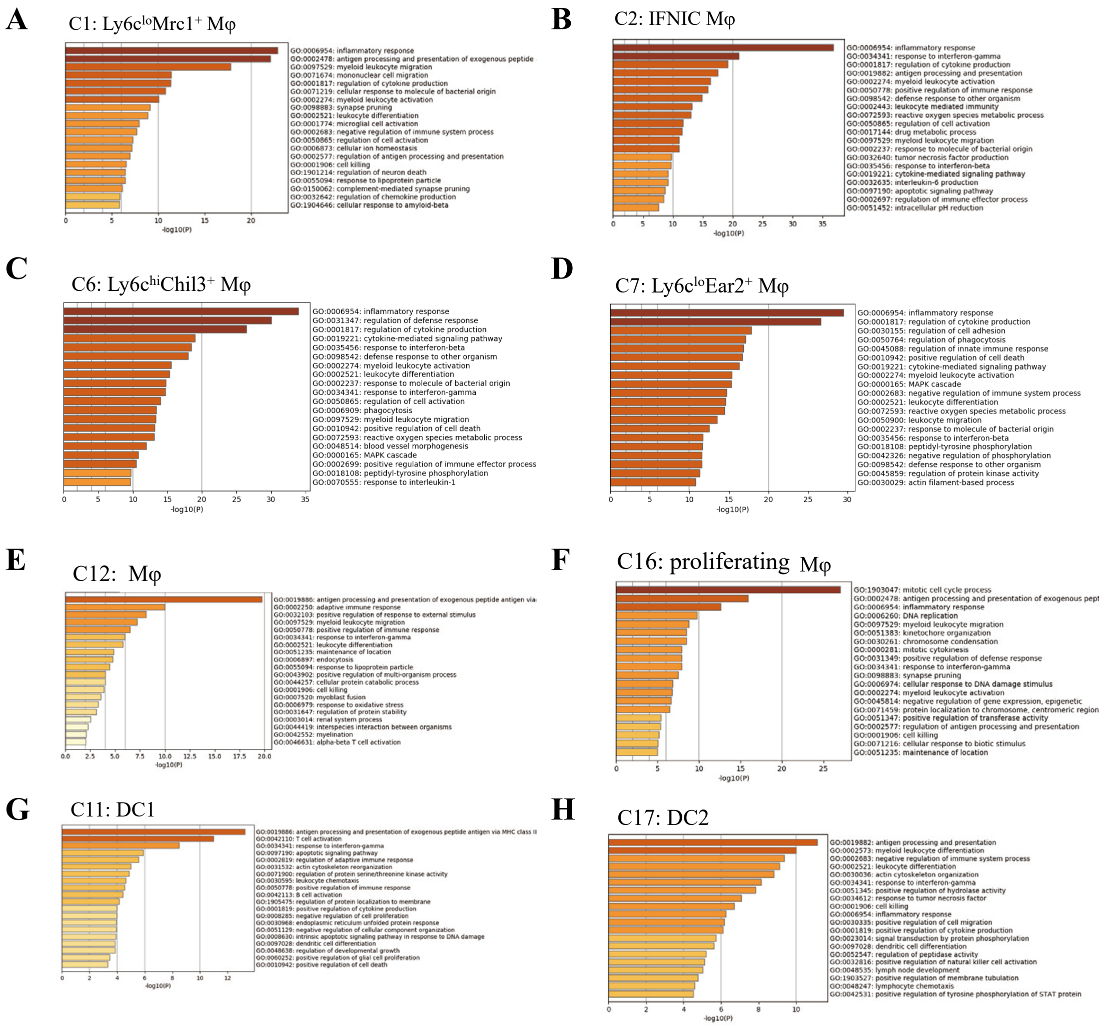

Supplementary Figure 5. Top 20 enriched gene ontology pathways in each macrophage cluster **(A-F)** and cDC cluster **(G, H)** as calculated by metascape. Only marker genes with avergage $log_2FC > 0.59$ were used for enrichment analysis. Mφ, macrophage; cDC, classical dendritic cell; FC, fold change.

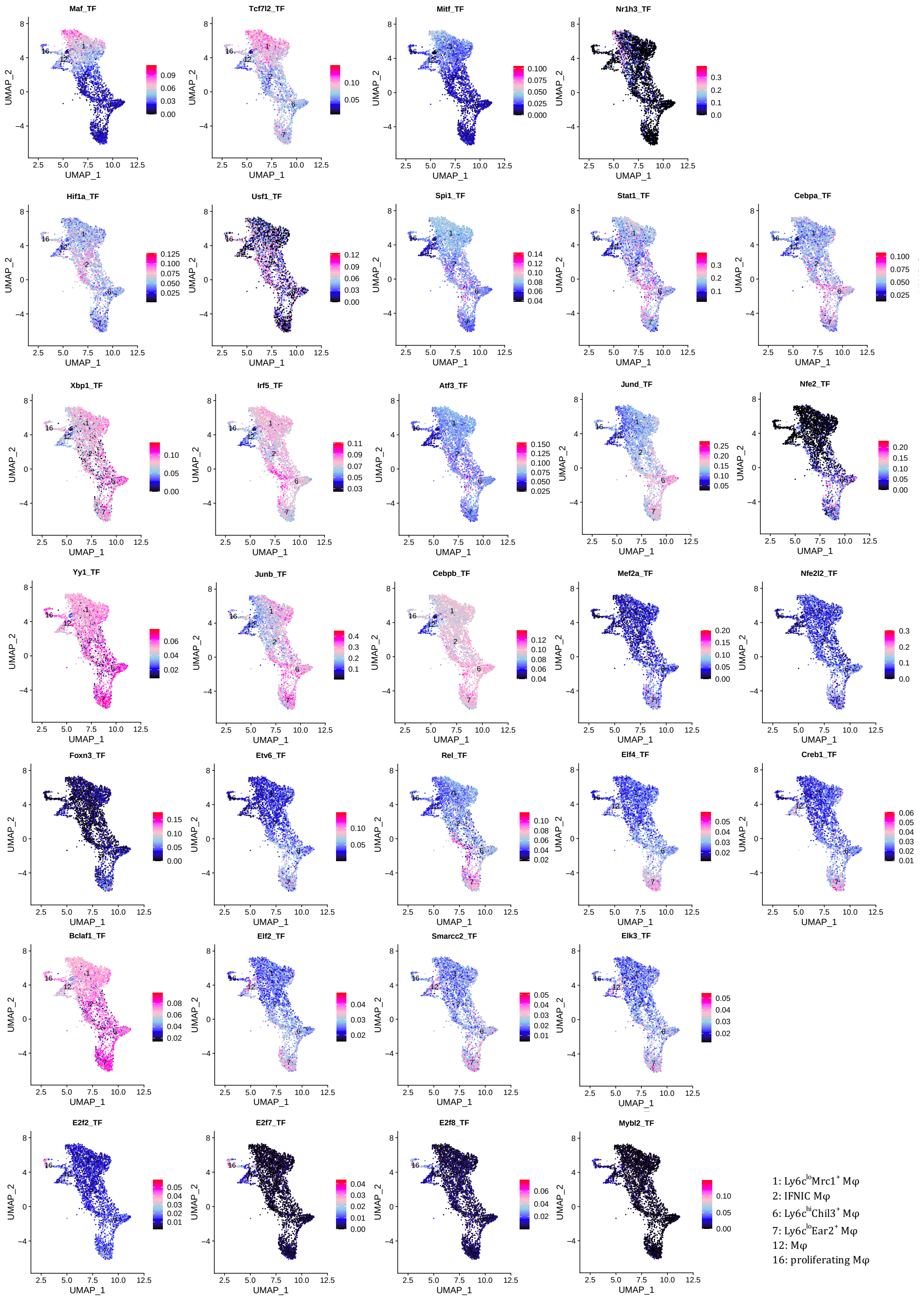

Supplementary Figure 6. Gene regulatory networks in macrophages subtypes. Featureplot of the area under the curve scores for the expression of gene sets regulated by transcription factors, as estimated with SCENIC, in each macrophage cluster. AUC score bar is shown on the right. Cell identities are annotated in the lower right corner SCENIC, single-cell regulatory network inference and clustering; AUC, area under the curve.

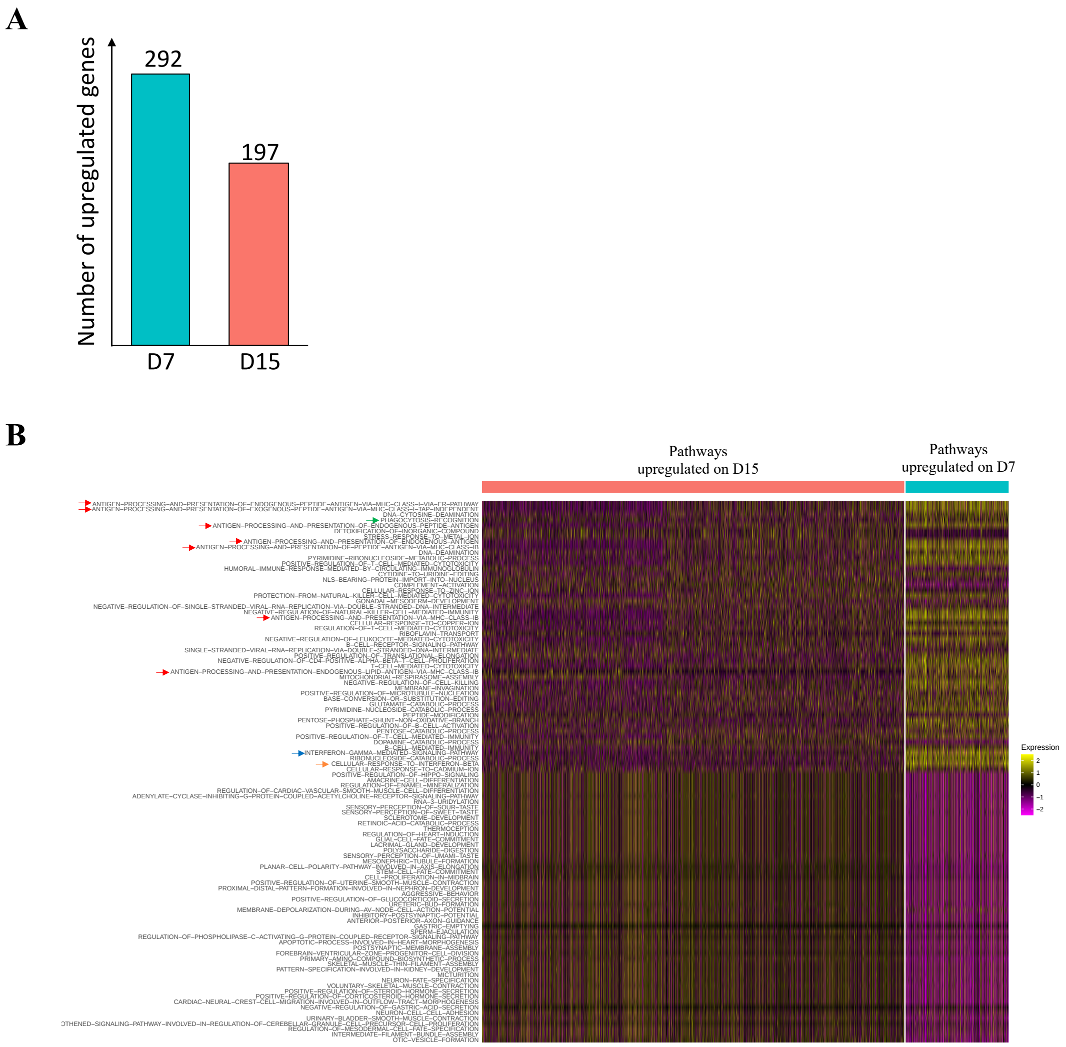

Supplementary Figure 7. Upregulated DEGs and top differential GO biological process terms between macrophages on D7 and D15. (**A**) Number of DEGs between macrophages on D7 and D15. Upregulated genes are defined as avg_log2FC > 0.59, adj_p value < 0.01. (**B**) Heatmap showing top differential GO biological process terms assessed through GSVA. Scaled GSVA enrichment score is shown on the right. Only top 100 differential GO terms are shown (log_2Fold Change cutoff = 0.32, adjusted P value Cutoff = 0.05). GO terms pointed out with red arrows are associated with antigen processing and presentation, GO term "phagocytosis recognition" is pointed out with green arrow, GO term "Interferon gamma mediated signaling pathway" is pointed out with blue arrow, whereas GO term "Cellular response to Interferon beta" is pointed out with orange arrow. D7, 7 days following transplantation; D15, 15 days following transplantation.

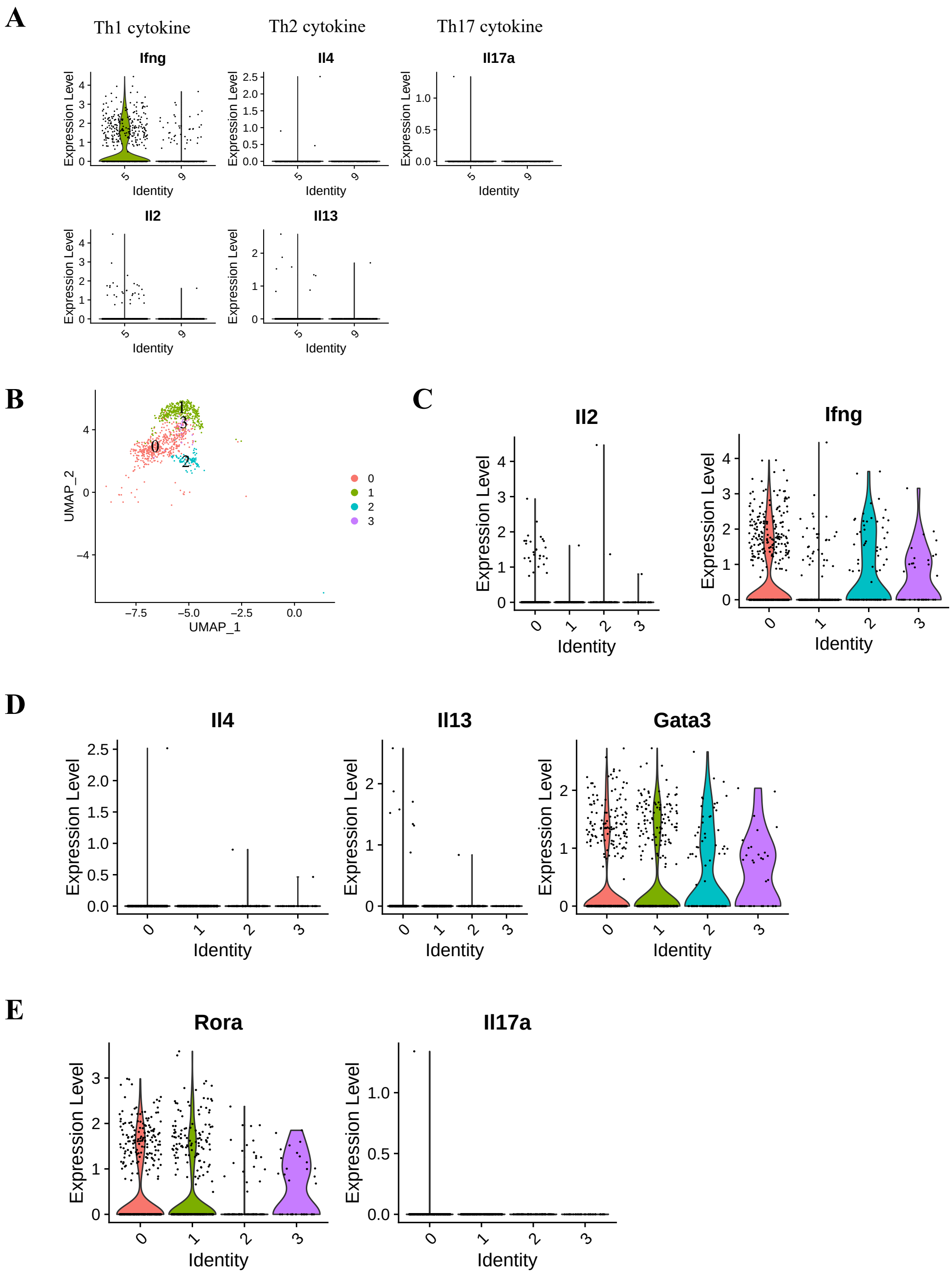

Supplementary Figure 8. **(A)** Violin plot showing normalized expression levels of the helper T cell markers in $CD4^{+}$ T cell subtypes. **(B)** Integrated umap plot of CD4 T cells re-clustered at higher resolution. Each dot depicts a single cell, colored according to cluster designation. Violin plots showing normalized gene expression level of **(C)** Th1, **(D)** Th2 and **(E)** Th17 markers.

A

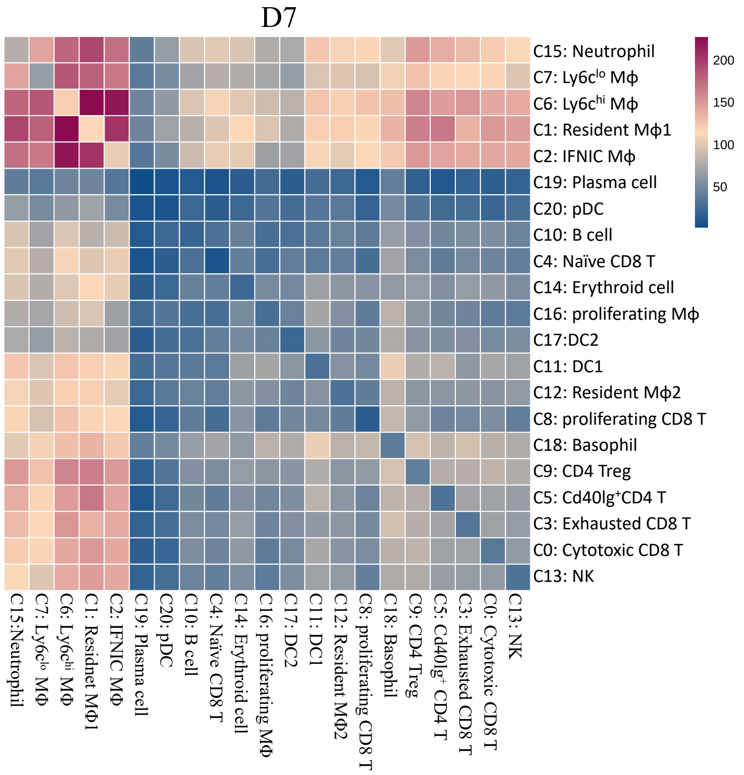

B

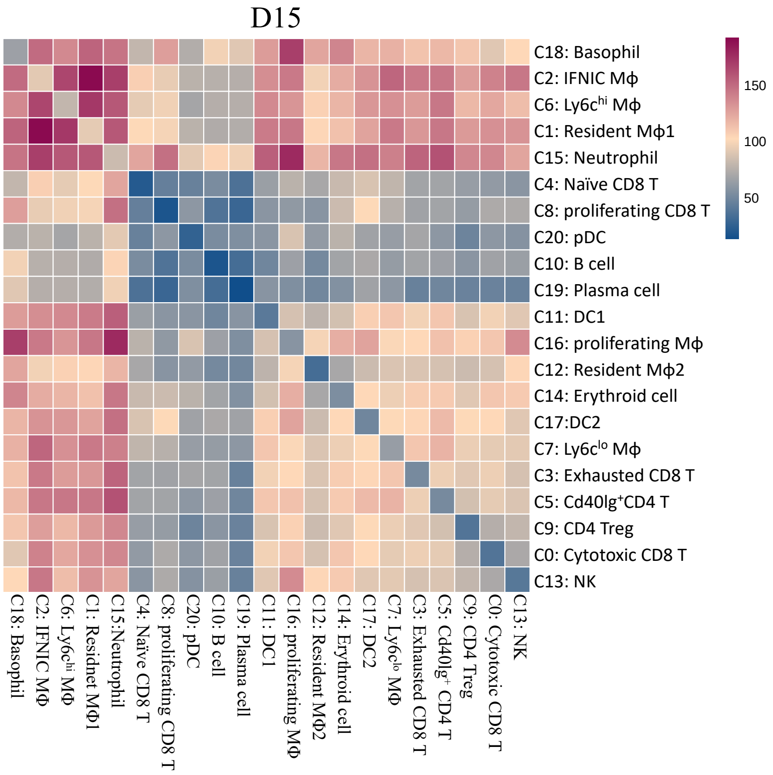

Supplementary Figure 9. Myeloid cells harbored the richest cell-cell interactions. Heatmaps showing capacity for intercellular communication between every two immune cell clusters on D7 **(A)** and D15 **(B)**. Color from blue to red indicates the number of LR pairs from low to high. D7, 7 days following transplantation; D15, 15 days following transplantation; LR, ligand and receptor.

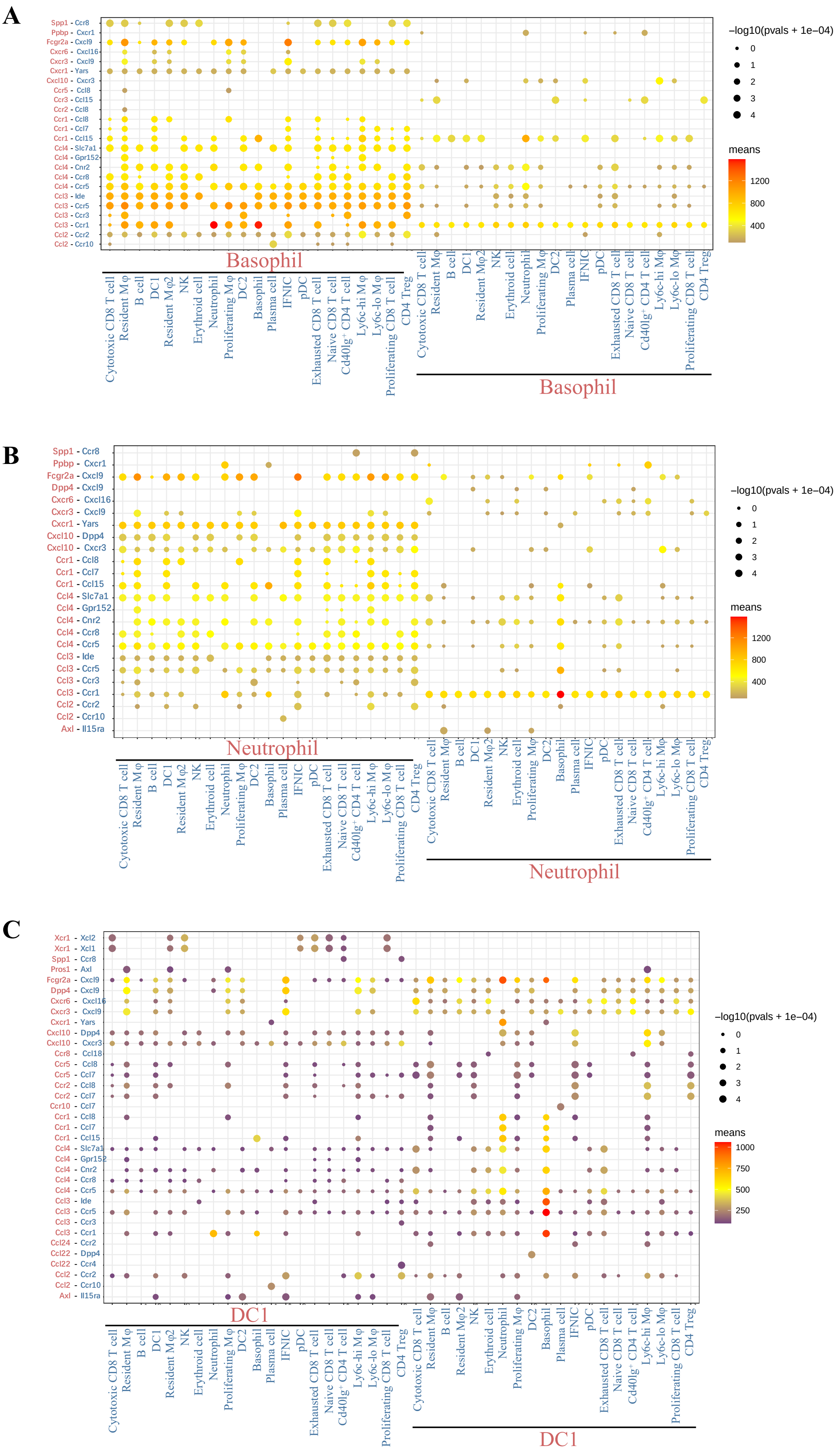
A
Spp1 - Ccr8
Ppbp - Cxcr1
Fcgr2a - Cxcl9
Cxcr6 - Cxcl16
Cxcr3 - Cxcl9
Cxcr1 - Yars
Cxcl10 - Cxcr3
Ccr5 - Ccl8
Ccr3 - Ccl15
Ccr2 - Ccl8
Ccr1 - Ccl8
Ccr1 - Ccl7
Ccr1 - Ccl15
Ccl4 - Slc7a1
Ccl4 - Gpr152
Ccl4 - Cnr2
Ccl4 - Ccr8
Ccl4 - Ccr5
Ccl3 - Ide
Ccl3 - Ccr5
Ccl3 - Ccr3
Ccl3 - Ccr1
Ccl2 - Ccr2
Ccl2 - Ccr10
Basophil
Cytotoxic CD8 T cell
Resident Mφ
B cell
DC1
Resident Mφ2
NK
Erythroid cell
Neutrophil
Proliferating Mφ
DC2
Basophil
Plasma cell
IFNIC
pDC
Exhausted CD8 T cell
Naive CD8 T cell
Cd40lg+ CD4 T cell
Ly6c-hi Mφ
Ly6c-lo Mφ
Proliferating CD8 T cell
CD4 Treg
Basophil
−log10(pvals + 1e−04)
0
1
2
3
4
means
1200
800
400
B
Spp1 - Ccr8
Ppbp - Cxcr1
Fcgr2a - Cxcl9
Dpp4 - Cxcl9
Cxcr6 - Cxcl16
Cxcr3 - Cxcl9
Cxcr1 - Yars
Cxcl10 - Dpp4
Cxcl10 - Cxcr3
Ccr1 - Ccl8
Ccr1 - Ccl7
Ccr1 - Ccl15
Ccl4 - Slc7a1
Ccl4 - Gpr152
Ccl4 - Cnr2
Ccl4 - Ccr8
Ccl4 - Ccr5
Ccl3 - Ide
Ccl3 - Ccr5
Ccl3 - Ccr3
Ccl3 - Ccr1
Ccl2 - Ccr2
Ccl2 - Ccr10
Axl - Il15ra
Neutrophil
Neutrophil
−log10(pvals + 1e−04)
0
1
2
3
4
means
1200
800
400
C
Xcr1 - Xcl2
Xcr1 - Xcl1
Spp1 - Ccr8
Pros1 - Axl
Fcgr2a - Cxcl9
Dpp4 - Cxcl9
Cxcr6 - Cxcl16
Cxcr3 - Cxcl9
Cxcr1 - Yars
Cxcl10 - Dpp4
Cxcl10 - Cxcr3
Ccr8 - Ccl18
Ccr5 - Ccl8
Ccr5 - Ccl7
Ccr2 - Ccl8
Ccr2 - Ccl7
Ccr10 - Ccl7
Ccr1 - Ccl8
Ccr1 - Ccl7
Ccr1 - Ccl15
Ccl4 - Slc7a1
Ccl4 - Gpr152
Ccl4 - Cnr2
Ccl4 - Ccr8
Ccl4 - Ccr5
Ccl3 - Ide
Ccl3 - Ccr5
Ccl3 - Ccr3
Ccl3 - Ccr1
Ccl24 - Ccr2
Ccl22 - Dpp4
Ccl22 - Ccr4
Ccl2 - Ccr2
Ccl2 - Ccr10
Axl - Il15ra
DC1
DC1
−log10(pvals + 1e−04)
0
1
2
3
4
means
1000
750
500
250

D

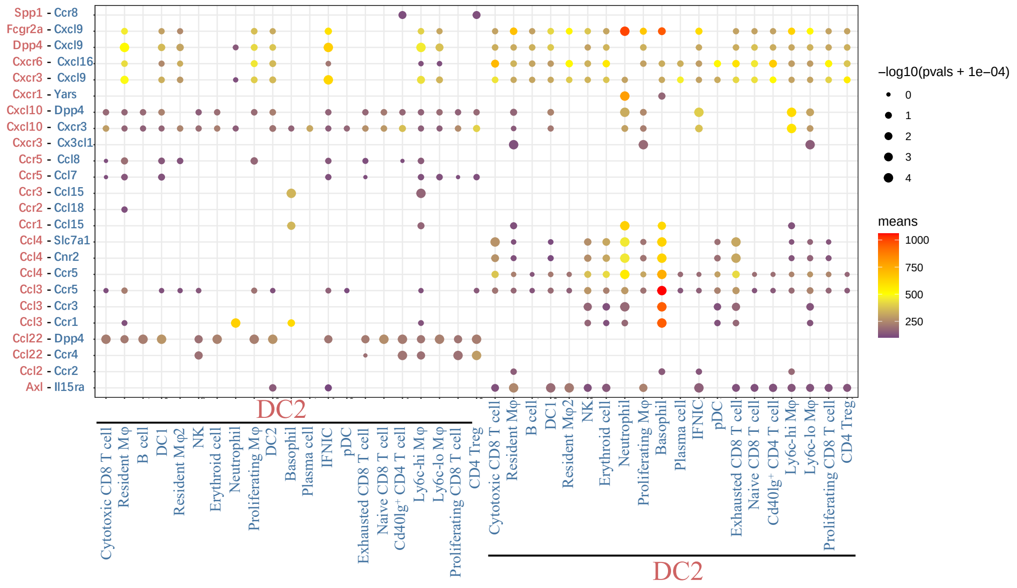

Supplementary Figure 10. (A-D) Overview of chemokine mediated LR interactions between granulocytes/DCs and other immune cell clusters. P values are indicated by circle size, with the scale to the right (permutation test). The means of the average expression levels of interacting molecule 1 in cluster 1 and interacting molecule 2 in cluster 2 are indicated by color. Assays were carried out at the mRNA level but were used to extrapolate protein interactions. Only LR pairs with means value > 100 are shown. DCs, dendritic cells; LR, ligand and receptor.

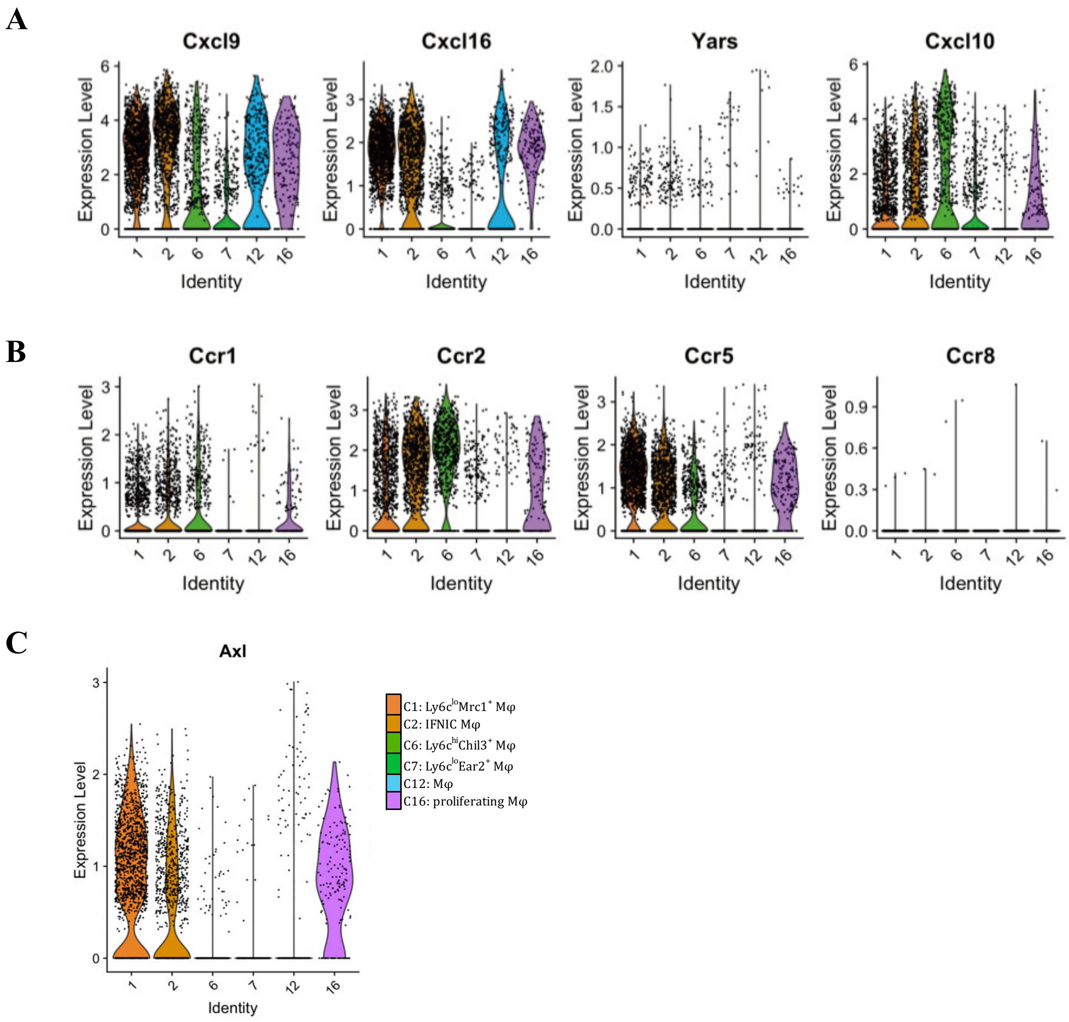

Supplementary Figure 11. Violin plot showing normalized gene expression level of **(A)** ligands Cxcl9, Cxcl16, Yars and Cxcl10, **(B)** receptors Ccr1, Ccr2, Ccr5 and Ccr8, **(C)** Axl in macrophage clusters. Mφ, macrophage.

Table S1. Summary of 20 major immune cell types in murine acute T-cell mediated kidney allograft rejection

Cell Cluster (Naming)	Marker genes	Transcription factors	Major functions	Count/Fraction of immune cells	
				Day 7 (3977)	Day 15 (6944)
Cluster 0 (cytotoxic CD8 T cell)	Cd8a, Cd8b1, Nkg7, Gzmb, Ifng, Cxcr6	Pbx1, Sp4, Usf2, Runx3	T cell activation, antigen receptor-mediated signaling pathway, interferon-gamma production	799 (20.09%)	1269 (18.27%)
Cluster 1 (Ly6c^{lo}Mrc1^{+} Mφ)	C1qa, C1qb, C1qc, Cd81, Adgre1, Apoe	Maf, Tcf7l2, Mitf, Nr1h3	Inflammatory response, macrophage activation antigen processing and presentation, receptor-mediated endocytosis, MAPK cascade, chemokine-mediated signaling pathway	167 (4.20%)	1309 (18.85%)
Cluster 2 (Ly6c^{inter} Mφ)	Aif1, Cxcl9, Ly6i, Saa3, Cfb, Lyz2	Usf1, Spi1, Hif1a, stat1	Inflammatory response, response to interferon-γ, antigen processing and presentation, reactive oxygen species metabolic process	283 (7.12%)	816 (11.75%)
Cluster 3 (exhausted CD8 T cell)	Cd8a, Cd8b1, Pdcd1, Lag3, Havcr2	Foxn2	T cell activation, adaptive immune response	473 (11.89%)	364 (5.24%)
Cluster 4 (Naive CD8 T cell)	Cd8a, Cd8b1, Tcf7, Lef1, IL7r, Ms4a4b	Runx1, Foxo1, Ets2, Elf1, Jun	Translation	446 (11.21%)	258 (3.72%)
Cluster 5 (Cd40lg^{+} CD4 T cell)	Cd4, Cd40lg, Icos, Tnfrsf18, Ifng	Gata3	T cell activation, regulation of cytokine production	272 (6.84%)	348 (5.01%)
Cluster 6 (Ly6c^{hi}Chil3^{+} Mφ)	Ly6c2, Chil3, Plac8, Hp, Ccr2, Vcan	Cebpa, Xbp, Irf5, Jund	Inflammatory response, response to interferon-β, phagocytosis, chemokine-mediated signaling pathway, would healing, cytokine-mediated signaling pathway	178 (4.48%)	430 (6.19%)
Cluster 7 (Ly6c^{lo}Ear2^{+} Mφ)	Ear2, Gngt2, Pou2f2, Nr4a1, Ace, Cx3cr1	Cebpb, Etv6, Rel, Elf4, Elf2, Creb1, Elk3, Smarcc2	Inflammatory response, cytokine-mediated signaling pathway, positive regulation of I-kappaB kinase/NF-kappaB signaling	110 (2.77%)	498 (7.17%)
Cluster 8 (proliferating CD8 T cell)	Cd8a, Cd8b1, Top2a, Mki67, Pclaf	E2f1, E2f2, E2f7, E2f8,	Mitotic cell cycle process	328 (8.25%)	122 (1.76%)
Cluster 9 (CD4 Treg)	Cd4, Il2ra, Foxp3, Ctla4, Il10	Stat5a, Tgif1, Cux1, Runx2	T cell activation, negative regulation of leukocyte activation, regulation of cytokine production	133 (3.34%)	279 (4.02%)

Cluster 10 (B cell)	Cd79a, Cd79b, Ly6d	Tgif1, Foxo1, Runx1, Jun	B cell activation, B cell differentiation, antigen processing and presentation	312 (7.85%)	96 (1.38%)
Cluster 11 (DC_1)	Cd209a, Flt3, Kit	Pbx1, Irf5, Hif1a, Cebpb	Antigen processing and presentation of exogenous peptide antigen via MHC class II, response to interferon-gamma	36 (0.91%)	322 (4.64%)
Cluster 12 (Mφ2)	C1qa, C1qb, C1qc, Cd81, Apoe, Aif1	Nr1h3	Antigen processing and presentation	91 (2.29%)	238 (3.43%)
Cluster 13 (NK cell)	Gzma, Klrb1c, Ncr1	Eomes, Irf8	Natural killer cell activation, regulation of tumor necrosis factor production	130 (3.27%)	141 (2.03%)
Cluster 15 (Neutrophil)	S100a9, Csf3r, Lcn2, S100a8, Retnlg, Ly6g	Stat1, Ets2, Cebpb	Inflammatory response, regulation of cytokine production, cytokine-mediated signaling pathway	152 (3.82%)	76 (1.09%)
Cluster 16 (proliferating Mφ)	Mki67, Top2a, C1qa, Aif1, Cx3cr1	E2f2, E2f7, E2f8, Mybl2	Mitotic cell cycle process, antigen processing and presentation	10 (0.25%)	148 (2.13%)
Cluster 17 (DC_2)	Fscn1, Cacnb3, Mreg, Flt3	Rel, Relb, Nfkb2, Stat5a	Dendritic cell differentiation, antigen processing and presentation, response to tumor necrosis factor	8 (0.20%)	92 (1.32%)
Cluster 18 (Basophil)	Mcpt8, Il6, Il4, Gata2	Gata1, Gata2, Fosl1	Inflammatory response, response to wounding, cytokine-mediated signaling pathway	9 (0.23%)	65 (0.94%)
Cluster 19 (Plasma B cell)	Jchain, Iglv1, Igkc, Iglc1, Ighg2b	Pbx1, Xbp1	Response to endoplasmic reticulum stress, immunoglobulin mediated immune response	5 (0.13%)	56 (0.81%)
Cluster 20 (pDC)	Siglech, Cox6a2, Klra17	Runx2	Cytoplasmic translation	35 (0.88%)	17 (0.24%)
CD8 T cells (cluster 0, 3, 4, 8)	…	…	…	51.44%	28.99%
Monocyte/Mφ (cluster 1, 2, 6, 7, 12, 16)	…	…	…	21.11%	49.52%

Mφ, macrophage; DC, dendritic cell; NK, natural killer; pDC, plasmacytoid dendritic cell.

Table S2. Genes used for pseudotime gene variation analysis

Gene Name	Gene Name	Gene Name
Cx3cr1	Tmem176a	Ly6c2
Selenop	Grn	Anax2
Vcam1	Ctsh	Myd88
Maf	Mif	Ifi47
Ccl7	Tubb5	Cxcl10
Cadm1	Cxcl9	Alox5ap
Adgre1	Ass1	Socs3
Mrc1	Cfb	Plaur
Apoe	Saa3	Plac8
Ctsb	Eno1	Ifitm3
Itm2b	Nme2	Ifitm2
Ccl8	Ccr2	Irf1
Lgmn	Cd40	Lsp1
Ckb	Isg15	Clec4e
Cd81	Upp1	Gpx1
Cd72	Il1rn	Nfkbia
Acp5	Prdx5	Pou2f2
Cd63	Sod2	Cytip
Fabp5	Ly6i	Ace
H2-M2	Zbp5	Klf2
Ccl12	F10	Gngt2
Mafb	Gbp2	Rel
Ctss	S100a10	Nr4a1
Ctsl	S100a6	Itga4
Cstd	S100a11	Btg1
C1qb	Tgtp1	Ear2
C1qc	Socs1	Atf3
C1qa	Fn1	Cybb
H2-Eb1	Sell	Il1b
Cxcl16	Chil3	Cebpb
Aif1	S100a4	Traf1
H2-Ab1	Crip1	Gsr
Cd74	Ms4a4c	Napsa
H2-Aa	Ifi205	Gpr141
Tmem176b		
